# Transitioning from wet lab to artificial intelligence: a systematic review of AI predictors in CRISPR

**DOI:** 10.1186/s12967-024-06013-w

**Published:** 2025-02-04

**Authors:** Ahtisham Fazeel Abbasi, Muhammad Nabeel Asim, Andreas Dengel

**Affiliations:** 1 Smart Data and Knowledge Services, German Research Center for Artificial Intelligence, 67663 Kaiserslautern, Germany; 2 Department of Computer Science, Rhineland-Palatinate Technical University Kaiserslautern-Landau, 67663 Kaiserslautern, Germany

**Keywords:** AI-driven CRISPR applications, Representation learning in CRISPR, ML/DL and CRISPR, CIRSPR on/off-target activity, CRISPR loci and operons, Anti-CRISPR proteins, Gene editing outcomes

## Abstract

The revolutionary CRISPR-Cas9 system leverages a programmable guide RNA (gRNA) and Cas9 proteins to precisely cleave problematic regions within DNA sequences. This groundbreaking technology holds immense potential for the development of targeted therapies for a wide range of diseases, including cancers, genetic disorders, and hereditary diseases. CRISPR-Cas9 based genome editing is a multi-step process such as designing a precise gRNA, selecting the appropriate Cas protein, and thoroughly evaluating both on-target and off-target activity of the Cas9-gRNA complex. To ensure the accuracy and effectiveness of CRISPR-Cas9 system, after the targeted DNA cleavage, the process requires careful analysis of the resultant outcomes such as indels and deletions. Following the success of artificial intelligence (AI) in various fields, researchers are now leveraging AI algorithms to catalyze and optimize the multi-step process of CRISPR-Cas9 system. To achieve this goal AI-driven applications are being integrated into each step, but existing AI predictors have limited performance and many steps still rely on expensive and time-consuming wet-lab experiments. The primary reason behind low performance of AI predictors is the gap between CRISPR and AI fields. Effective integration of AI into multi-step CRISPR-Cas9 system demands comprehensive knowledge of both domains. This paper bridges the knowledge gap between AI and CRISPR-Cas9 research. It offers a unique platform for AI researchers to grasp deep understanding of the biological foundations behind each step in the CRISPR-Cas9 multi-step process. Furthermore, it provides details of 80 available CRISPR-Cas9 system-related datasets that can be utilized to develop AI-driven applications. Within the landscape of AI predictors in CRISPR-Cas9 multi-step process, it provides insights of representation learning methods, machine and deep learning methods trends, and performance values of existing 50 predictive pipelines. In the context of representation learning methods and classifiers/regressors, a thorough analysis of existing predictive pipelines is utilized for recommendations to develop more robust and precise predictive pipelines.

## Introduction

According to the World Health Organization (WHO), more than 10,000 diseases have emerged with unique characteristics, causes, and symptoms [32]. These diseases can be placed into four categories namely, infectious, non-communicable, genetic, and others [16, 67, 89]. Infectious diseases are caused by microorganisms such as bacteria, viruses, or parasites and can spread directly or indirectly from one person to another [16]. Genetic diseases arise from mutations or alterations in an individual’s DNA. These mutations can be inherited from parents or occur spontaneously [89]. Non-communicable diseases arise from genetic, physiological, environmental, and behavioral factors [69]. These chronic conditions develop gradually and are not typically passed from person to person [67]. Additionally, other diseases include various conditions arising from different mechanisms, such as injuries caused by external factors and congenital anomalies resulting from developmental malformations.

Among the four major disease categories, infectious and non-communicable diseases have a wider range of treatment possibilities. These options encompass traditional medications and cutting-edge therapies, including Proteolysis Targeting Chimeras (PROTACs) and RNA-based approaches [189]. However, these treatments are often ineffective for genetic diseases such as cancers, autoimmune disorders, hereditary conditions, and nervous system disorders [184]. This is because these diseases involve complex genetic mutations and pathways which are not easily targeted by conventional or even some modern therapies. Additionally, the variability in genetic makeup among individuals can result in diverse responses to treatment, making it difficult to develop universally effective therapies.

Following the need of a more effective treatment for genetic diseases, Doudna et al. [11, 45] proposed a unique system for DNA sequence editing named Clustered Regularly Interspaced Short Palindromic Repeats (CRISPR). CRISPR offers multiple advantages including, speed, flexibility, cost-effectiveness, and the capacity to manipulate multiple genomic locations simultaneously [4, 77]. The effectiveness of CRISPR system has been proven by various clinical studies related to diseases such as inherited eye disease Leber congenital amaurosis (LCA), Duchenne muscular dystrophy (DMD), and genetic lung and liver diseases [165]. In addition, the CRISPR system has been approved as the first gene therapy for Hemoglobinopathies (sickle cell disease) [36].

**Fig. 1:**
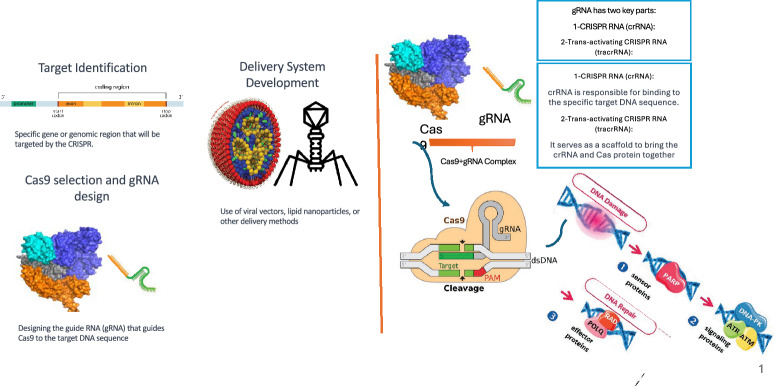
Adaptation of CRISPR system for DNA editing. In the very first step, target regions are identified with the selection of an appropriate CRISPR system. Afterward, CRISPR, trans-activating, and gRNA are designed to help Cas proteins cleave at the desired site. The complete complex is then delivered inside the cell with the help of a vector (virus)

Figure 1 graphically represents a generic muti-step process of CRISPR for the treatment of genetic diseases [207]. The multi-step process initiates with the identification of problematic regions in the DNA such as disease-related genes identification [114]. Following characteristics of desired cleavage regions, the next step is to design a CRISPR system which includes deep analysis of available diverse types of Cas Proteins, and design of guide RNA. gRNA is itself made up of two different parts i.e., CRISPR RNA, guides the Cas protein to the specific location in the genome where the cut is to be made, and trans-activating RNA which is necessary for binding of crRNA and the Cas protein, helping to form the active CRISPR-Cas9 complex [130]. The active CRISPR complex is sent into living organisms through a delivery system such as viral mechanisms or lipid nanoparticles [114]. In the CRISPR complex, guide RNA contains instructions about cleavage regions and Cas proteins cleave those regions. Sometimes this complex does not make cleavage at desired locations due to multiple reasons such as the weak design of guide RNA, off-target effects, poor delivery efficiency, chromatin accessibility, and cellular repair mechanisms. To make sure whether the designed complex will make cleavage at desired locations or not, researchers perform a deep analysis of this complex with the possibility of making cleavage at desired locations or wrong locations. Furthermore, if the CRISPR complex initiates cleavage at incorrect positions, a specialized process can be employed to halt it by introducing anti-CRISPR (acr) proteins and anti-CRISPR-associated proteins [225], which inhibit the CRISPR complex from making further cuts in the DNA. Finally, after DNA is cleaved, it has natural processes to repair the cleaved DNA with two different types of DNA repair mechanisms i.e., Non-Homologous End Joining (NHEJ), and Homology-Directed Repair (HDR) [134]. After, this rebuilding process there is a need to perform a genetic analysis of mutations. In a nutshell, this whole process contains 10 distinct types of tasks including CRISPR arrays [37], CRISPR loci [157], CRISPR-Cas systems [145], acr proteins and their activity [108], aca proteins [216], CRISPR operons [217, 223], Cas protein [235], Off-target activity [21], On-target activity [94], editable target regions [10], and gene editing outcomes [116]. A brief biological foundation of all 10 tasks is given in section .

Notably, all these tasks are usually performed through wet-lab experiments that are expensive, time-consuming, and error-prone. Following the success of artificial intelligence (AI) in diverse fields and with an aim for transitioning from wet-lab to AI-driven applications for CRISPR-based therapies development, researchers are trying to develop AI-driven applications for all 10 tasks [21, 35, 94, 116, 145, 223]. Although several AI-driven applications have been developed for CRISPR systems there is still a lot of room for the development of new applications. To accelerate and expedite the development of AI-driven applications for all 10 tasks, apart from the development of task-specific applications, in the last 3 years, 13 review articles have been published [13, 41, 64, 76, 84, 93, 99, 144, 151, 162, 164, 177, 194]. The primary focus of these articles was to summarise existing AI predictors within the context of CRISPR. The focus of these review articles is often constrained to only a single task of CRISPR and fail to bridge the gap between the broader landscape of CRISPR and AI predictors trends.

With an aim to bridge the gap between both fields and provide a unique platform encompassing biological foundations and AI advancements related to all 10 tasks, the contributions of this manuscript are manifold. 1) First, it equips AI researchers with biological foundations of 10 distinct tasks of CRISPR. 2) It presents details of the existing 80 public datasets related to 10 distinct tasks and provides overview of 10 public CRISPR databases for the development of new datasets. 5) In the context of all 10 tasks, it provides an in-depth analysis of the representation learning methods and classifiers/regressors employed in the existing AI predictors. 6) It discusses experimental settings and evaluation strategies utilized to evaluate existing AI-driven applications across 10 distinct tasks. 7) Finally, it provides performance values of 50 predictive pipelines across 80 public benchmark datasets of 10 distinct tasks. AI researchers can utilize this information to find predictors’ architectural details and current state-of-the-art performance values of predictors for each task.

## Examining CRISPR tasks through the lens of AI researcher

**Fig. 2:**
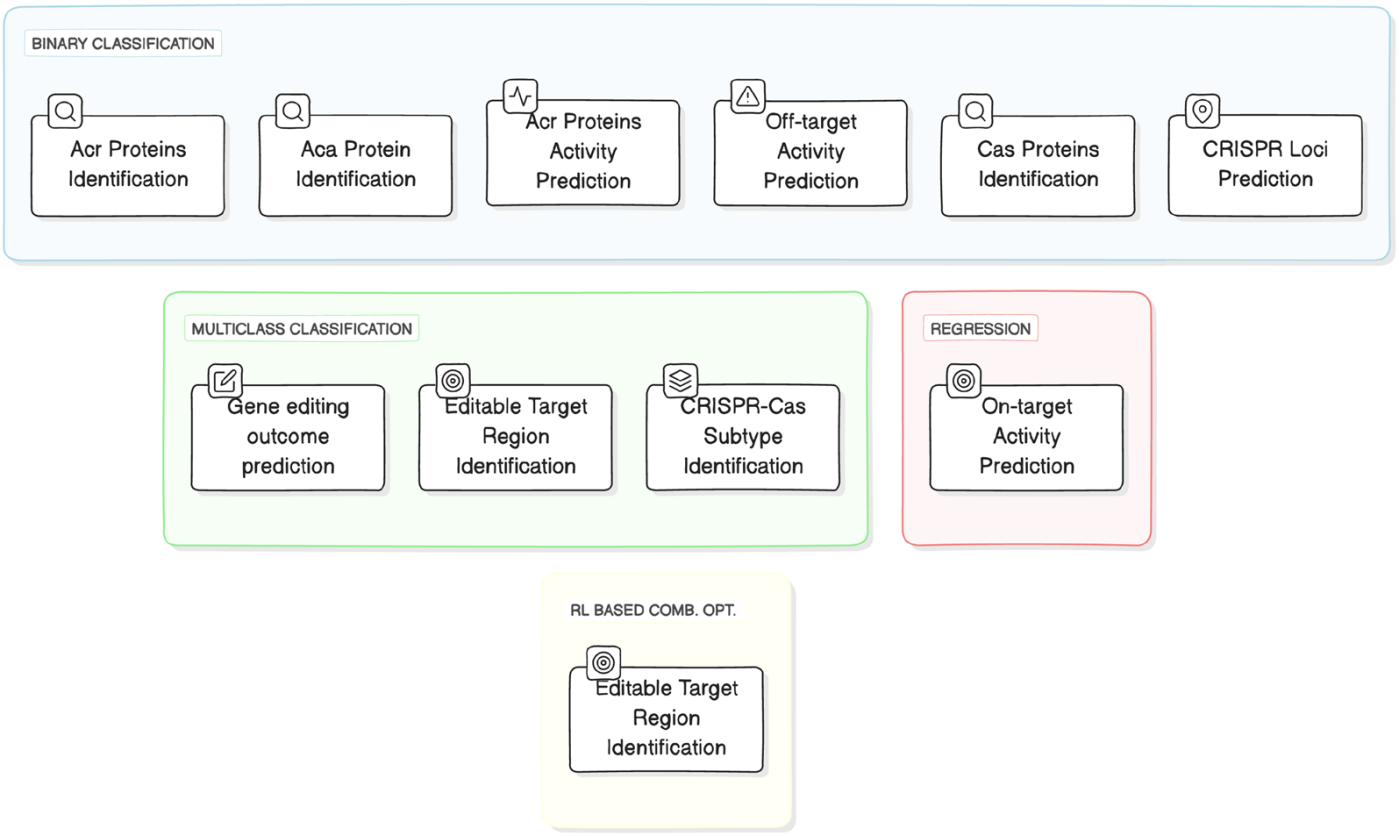
Categorization of CRISPR related applications/tasks

AI researchers often lack a deep understanding of the biological foundations of CRISPR and generally show little interest in the development of AI-driven CRISPR applications. In addition, the alignment of CRISPR tasks with AI paradigms may require extensive effort for understanding CRISPR tasks background knowledge. However, AI researchers can be facilitated by aligning CRISPR tasks with their familiar AI paradigms-such as binary classification, multi-class classification, or regression. In this context, we aligned 10 different CRISPR-related tasks from an AI perspective with their associated AI paradigms. Figure 2 visually illustrates 10 CRISPR tasks alignment with 4 AI paradigms namely binary classification, multi-class classification, regression, and reinforcement learning (RL) based optimization.

**Fig. 3:**
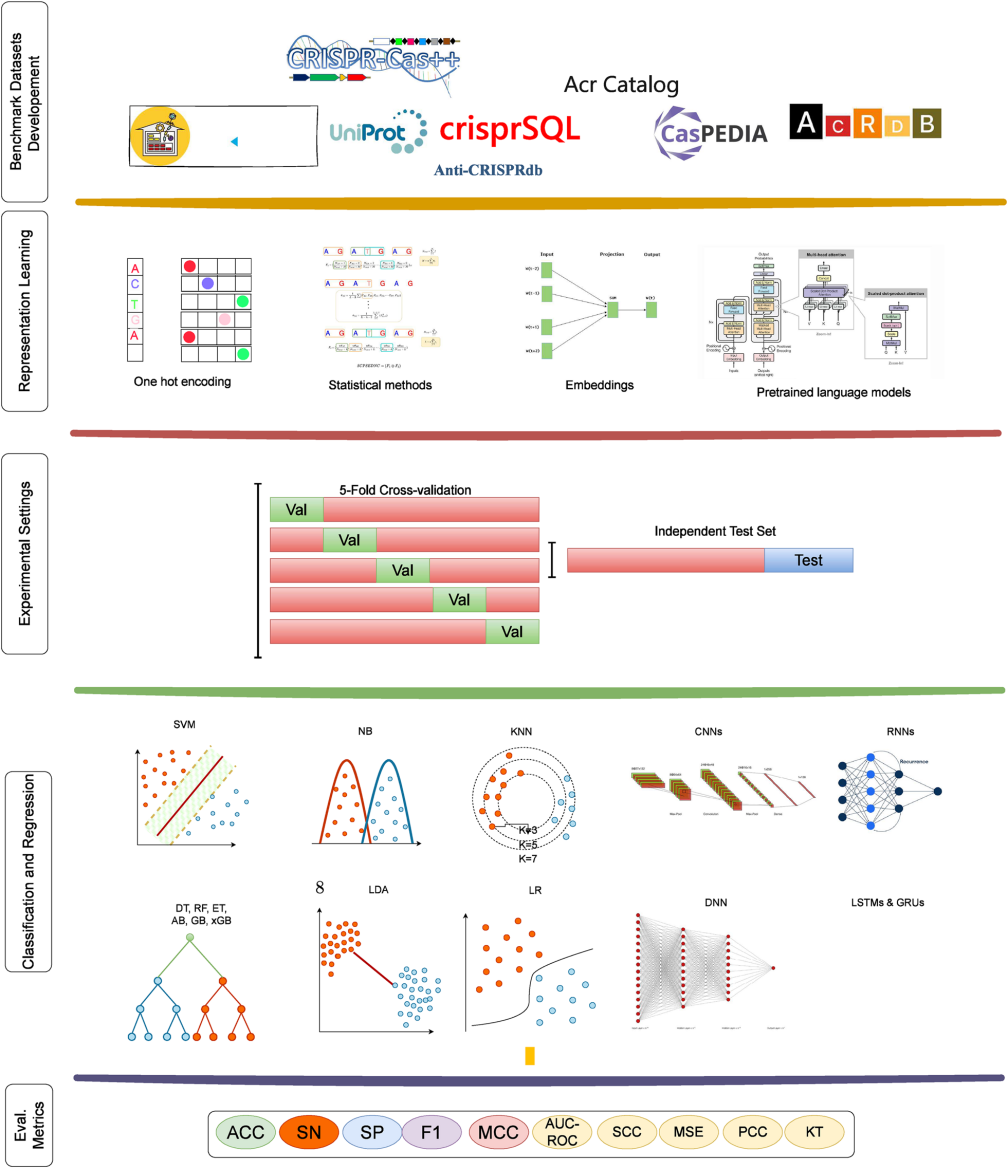
Components of AI predictive pipelines for CRISPR tasks involve several key steps. Initially, datasets are generated from publicly available databases. These sequences are then transformed into numerical vectors using statistical methods, one-hot encoding (OHE), or embedding techniques. The resulting datasets are divided into training and testing sets through cross-validation or independent testing. Subsequently, AI predictors are trained and tested, with evaluation scores calculated to determine their predictive performance

Figure 3 provides an overview of AI predictive pipeline for all 4 paradigms. This pipeline begins with the creation of novel benchmark datasets from publicly available sources. A high level analysis of Fig. 3 reveals that within aforementioned 4 AI-paradighms development of AI-driven CRISPR applications comprises 4 different components. These components include the development or utilization of existing benchmark datasets, transformations of raw sequences into numerical vectors, utilization of classifier or regressor, and evaluation measures. The datasets are usually developed by acquiring sequences and associated information from public databases. Detailed information about commonly used CRISPR databases and existing benchmark datasets can be found in sections and . Sequence data cannot be directly used in ML and DL classifiers or regressors due to their dependence on numerical vectors, representation learning methods are employed to convert sequences into numerical vectors. Section  elaborates distinct types of sequence representation learning methods that are employed in existing AI-driven CRISPPR applications. ML and DL predictors that are utilized in existing AI-driven CRISPR applications are described in section . Finally, in order to assess predictive pipelines, numerous evaluation measures are used which are comprehensively discussed in section .

### Existing reviews

To enhance the integration of AI approaches into CRISPR, 13 distinct review articles have been published in the last four years. The primary focus of these articles is to summarise insights of existing AI predictors that have been developed to empower CRISPR. Table 1 provides a comparative overview of these reviews, including the number of research articles they covered, their overall scope, and limitations.

A high-level analysis of the scope of 13 existing review articles in Table 1 reveals that these articles can be categorized into three distinct groups based on their focus on AI predictors for CRISPR. Two reviews delve exclusively into ML driven CRISPR methods [144, 151], while another two explore DL methods [93, 99]. Notably, the remaining nine reviews covered both ML and DL applications within CRISPR [13, 41, 64, 76, 84, 164, 177, 194].

Leveraging insights from Fig. 5 and the CRISPR summarized in the introduction section, a comprehensive review article should focus on the analysis of developed AI-driven applications for 10 distinct CRISPR tasks. The focused CRISPR tasks are on/off-target activity prediction [21, 94, 232], CRISPR array [37], loci, and system identification [145], acr and aca prediction [109], acr activity prediction [35], gene editing outcome prediction [23], and CRISPR operons identification [100]. Within this scope, an in-depth analysis of existing review articles reveals that 8 review papers emphasize on the design of CRISPR systems and the on/off-target activity prediction combined with additional topics such as cancer treatment or the usage of nanovectors [13, 64, 84, 93, 144, 164, 177, 194]. Two review papers incorporate 3 different tasks including acr proteins, gene editing outcomes, Cas9 activity [41, 99]. One review focuses on drug discovery [84] and one combines CRISPR and the development of biosensors [76]. It is noteworthy to mention that, out of all review papers, only Sharma et al. [162] cover 6 distinct tasks related to CRISPR i.e., PAM prediction, gRNA designing, on/off-target activity prediction, and Prime editing and pegRNA designing.

The overall objective of existing review papers was to consolidate AI predictors related to all tasks of CRISPR into a single platform. Additionally, these review papers attempted to bridge the gap between AI researchers and the complex biology associated with the various facets of CRISPR. However, there are two significant problems with the existing review papers. First, none of the review papers provide a complete and comprehensive picture of all CRISPR-related tasks. Secondly, these reviews inadequately capture the current landscape of datasets, feature extraction methods, and ML and DL models across various CRISPR tasks. Consequently, the bridge between AI and CRISPR remains incomplete due to the limitations of the current literature. For example, Wang et al. [194] offers a survey of ML and DL models used to predict CRISPR gRNA on and off-target activities. However, they only examine 10 distinct models for this purpose and do not delve deeply into trends regarding datasets and feature representation methods. Furthermore, they neglect other important aspects of CRISPR, such as gene editing outcomes, aca and acr proteins prediction. Dimarou et al. [41], focus on creating a catalog of ML and DL applications in CRISPR/Cas9 gRNA design, without delving into the specific technical details of ML/DL methods. Similarly, Khoshandam et al. [84] offer a generic perspective on the applications of AI in CRISPR. Particularly, only Lee et al. [99] and Sharma et al. [162] delve into the diverse applications of DL across different aspects of CRISPR, including on/off-target activity, automated systems, Cas9 variants, PAM prediction, and peGRNA design. However, both of these articles also fail to capture the current trends in feature extraction methods and AI algorithms used in CRISPR research. Moreover, crucial topics like CRISPR arrays, operons, and aca proteins are conspicuously absent. Furthermore, details regarding the datasets used in the research articles are rarely discussed. This limited scope hinders a comprehensive understanding of the current landscape of AI predictors in CRISPR research. While existing review articles provide some overview of CRISPR-associated predictive pipelines, there is a pressing need to consolidate diverse information into a unified platform that offers comprehensive insights, patterns, and trends in CRISPR-associated predictive pipelines.

## Methodology

**Table 1:** CRISPR and Machine Learning/Deep Learning related reviews

Article	Year range	Papers	Scope	Shortcomings
[41]	2017–2022	57	This study focuses on ML techniques to predict CRISPR/Cas9 sgRNA activity (on/off-target cleavage), to assist sgRNA design and identify current research trends.	The study is limited to a systematic mapping, excluding comparisons of methods or results.
[99]	2019–2023	54	This review article focuses on the applications of DL in multiple aspects of CRISPR-Cas, the prime focus is on gRNA activity prediction, CRISPR-Cas editing outcomes, design of High-Activity gRNAs, Automated System Implementation, Nucleic Acid Detection, Anti-CRISPR Protein Identification, Cas9 Variant Activity Prediction, Transcription Factor Binding Prediction	Not all topics are equally focused on. ML models, feature representation methods, and publicly available CRISPR-Cas associated benchmark datasets are not discussed.
[76]	2016–2019	11	Future of CRISPR-based biosensors, genome engineering, discovery of CRISPR, conventional biosensors, IoT, Big Biomedical Data, Cloud Computing Systems, integration of AI in CRISPR-based biosensors	There is no discussion on the use of AI in CRISPR.
[13]	–	–	Applicability of CRISPR/Cas9 in cancer research, CRISPR/Cas9 in drug resistance, CRISPR clinical trials, on/off-target gRNA activity prediction	The focus in biological/biochemical aspects is much bigger than on AI
[151]	till 2022	–	ML models in cancer, limited CRISPR details, drug discovery through AI/ML, precision and genomic medicine, different ML Models	Deep Learning is not described in detail and there is only a small dicussion of CRISPR
[177]	2014–2022	15	CRISPR for breast cancer treatment, AI/ML for therapy strategy, on/off-target effects of gRNA	Specific focus on Triple Negative Breast Cancer, no other fields than on/off target effects are dealt with
[84]	2017–2022	21	A perspective on AI in CRISPR/Cas9 modification, gRNA design, clinical trials. It explores how AI can enhance CRISPR’s precision and effectiveness in treating genetic diseases, particularly cancer, while also examining the current limitations and future possibilities of this approach.	This perspective study does not discuss any details of benchmark datasets, feature engineering approaches, and ML or DL methods.
[144]	–	-	ML effects on CRISPR gene editing, data labeling pitfalls, data selection, feature engineering, gRNA design and effects prediction	Only on/off-target activity prediction is discussed
[164]	2014–2022	49	ML/DL models in CRISPR/Cas9, on/off target activity prediction, data preprocessing, gRNA encoding	Only on/off-target activity prediction is discussed
[64]	2017–2021	9	AI in designing gene delivery vehicles, improving CRISPR/Cas, nanobots and mRNA vaccine carriers develpment	No other fields than on/off target effects are dealt with
[93]	2015–2021	20	On-target activity prediction, gRNA design, DL tools evaluation, comparison of learning based (DL) and hypothesis driven tools	No other fields than on target effects are dealt with
[194]	till 2019	20	ML/DL algorithms for on/off target prediction, gRNA design, challenges in CRISPR activity and specificity prediction	No other fields than on target effects are dealt with
[162]	till 2023	–	ML and DL models in PAM prediction, gRNA designing, on/off-target activity prediction, and Prime editing and pegRNA designing	Important details related to datasets, representation learning, and ML and DL models are missing. In addition, only 5 different of AI in CRISPR are covered.

**Fig. 4:**
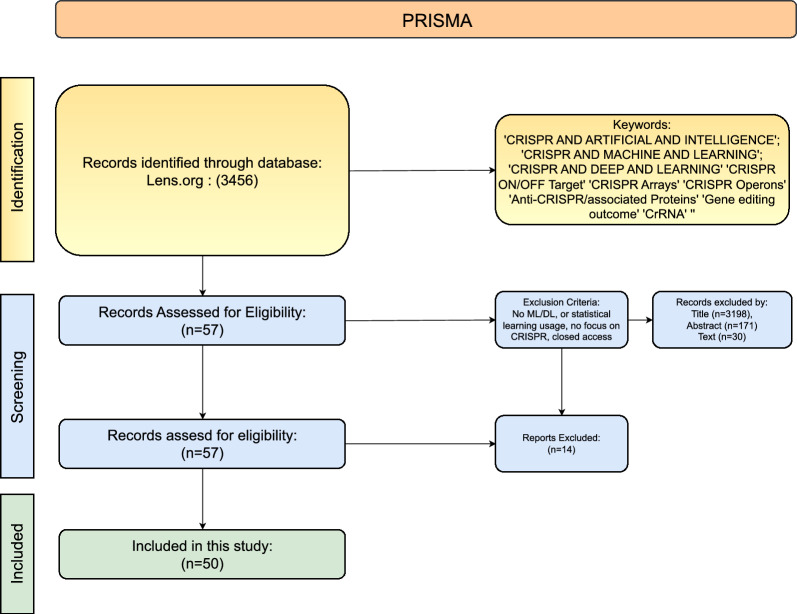
This figure depicts the workflow of searching and screening articles, with ’n’ representing the number of papers at each stage

This section explains different stages of preferred reporting items for systematic review and meta-analysis (PRISMA) strategy [161], which is used to gather relevant papers on the applications of AI in different CRISPR tasks. Figure 4 illustrates a visual representation of various stages from PRISMA that are summarized in the following subsections.

### Search strategy

Figure 4 illustrates the identification stage with different combinations of keywords that are utilized to search research articles. The keyword block has two key operators i.e. ’AND’ and ’OR’. We leverage these operators to connect keywords and build search queries such as, *’CRISPR AND ARTIFICIAL INTELLIGENCE’*, *’CRISPR AND MACHINE LEARNING’*, *’DEEP OR MACHINE AND LEARNING AND EFFICACY AND CRISPR’* and *’MACHINE OR DEEP LEARNING AND CRISPR’*. These queries are utilized in literature search engines like Lens (https://www.lens.org/) and Google Scholar (https://scholar.google.com/) for literature searches from Jan 2020 to Dec 2023. With the help of these queries, a substantial number of 3456 research articles are retrieved which are screened further.

### Screening strategy

With an aim to retain papers related to CRISPR-Cas, title, and abstract-based screenings are carried based on following criteria:

- Studies, that make use of ML or DL techniques.
- Studies exclusively focus on CRISPR problems.
- Studies with open access.

Following the preliminary title based screening, 3198 papers are sifted out. In a subsequent step, these 258 papers are screened by abstract, resulting in 87 papers for full-text screening. An additional 27 papers are discarded after scrutinizing the full text of the papers. After thoroughly reviewing the text of the papers, 50 papers are ultimately selected for the literature review.

##  Background of CRISPR tasks and benchmark datasets for development of AI predictive pipelines

**Table 2:** Problem distribution of reviewed papers

Problem	Count	Reference
CRISPR arrays	2	[37, 128]
CRISPR loci	2	[137, 157]
CRISPR systems CrRNA	2	[145, 147]
Acr proteins	6	[35, 47, 57, 109, 193, 242]
Aca proteins	1	[216]
CRISPR operons	2	[217, 223]
Cas protein	2	[220, 235]
Off target activity	10	[78, 112, 117, 139, 172, 178, 187, 221, 232, 234, 238]
On target activity	16	[34, 40, 50, 61, 92, 103, 127, 138, 141, 152, 209, 219, 228, 231, 232, 234]
Editable target region	1	[10]
Gene editing outcome prediction	4	[6, 105, 116, 163]
Acr proteins activity	2	[63, 132]

This section provides a concise overview of 10 distinct CRISPR tasks. These tasks encompass on/off-target activity prediction, acr proteins prediction, gene editing outcome prediction, CRISPR arrays analysis, acr associated proteins prediction, and Cas proteins identification. Additionally, it presents sample statistics and details of various public benchmark datasets pertinent to each task, facilitating the development of innovative AI tools. This section also discusses the distribution of AI predictors for each task and the types of datasets utilized in their experimental setups.

### Basics of CRISPR

**Fig. 5:**
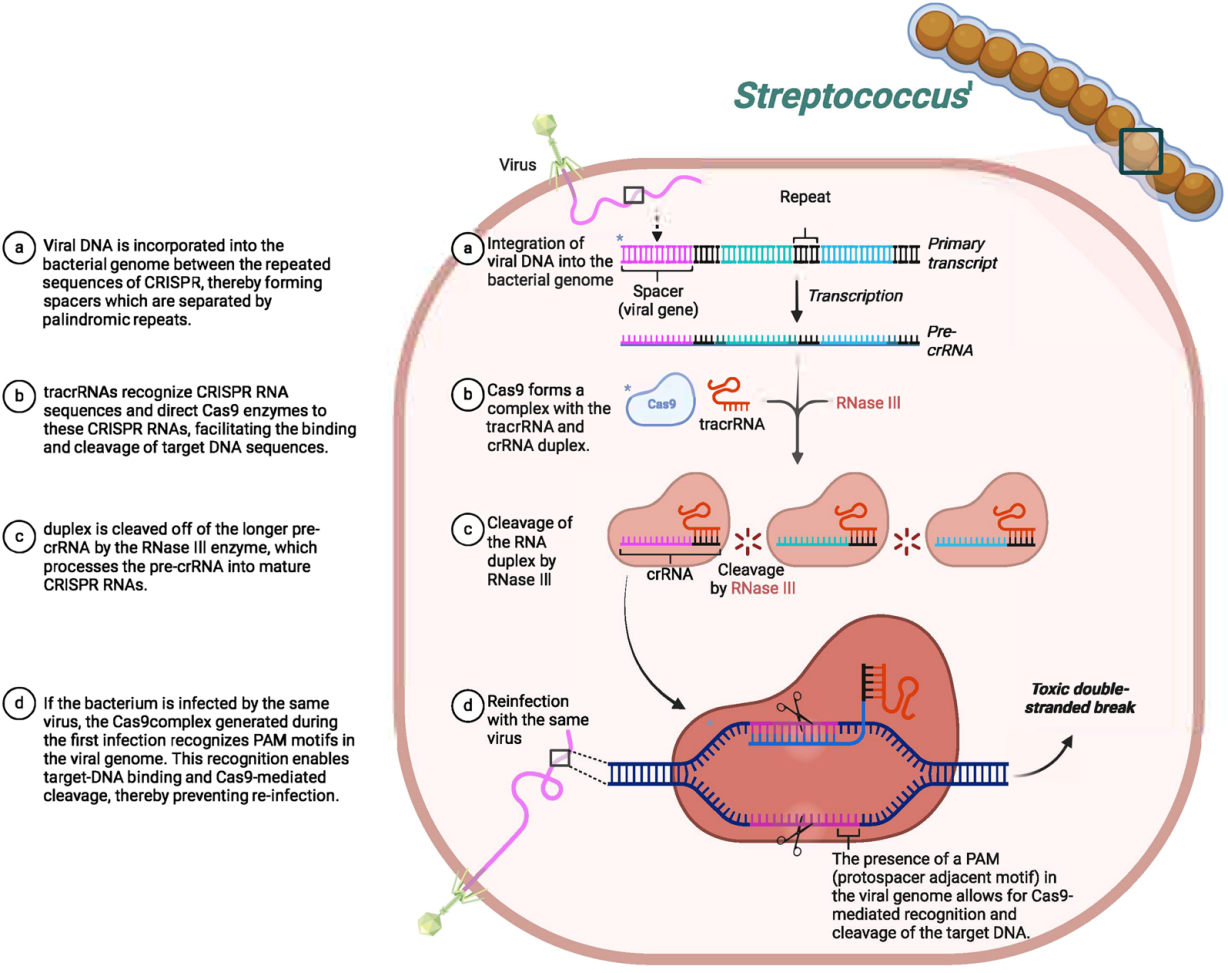
Viral DNA is integrated into the bacterial genome, forming spacers. tracrRNAs guide Cas9 to process and mature crRNAs. Cas9, directed by crRNAs, cleaves viral DNA upon reinfection, preventing further infection. (Image created using Biorender.com)

CRISPR originates from bacteria and develops inside the bacterial genome as a defense mechanism through past encounters with the foreign genetic material of viruses or plasmids [70, 79]. The overall process of viral infection and bacterial response is shown in Fig. 5. Particularly, this bacterial defense mechanism against viral sequences is adopted in genetic engineering, where synthetic guide RNAs (gRNAs) are designed and coupled with bacterial Cas proteins for genome editing purposes [70].

The foundation of CRISPR system is based on a CRISPR array, consisting of three essential components: a leader sequence, repeats, and spacers [170]. Leader sequence is short and non-repetitive promoter like sequence that helps to initiate the process of transcription in a CRISPR array [5]. Spacers are short repetitive sequences that are incorporated from the viral genome and repeats are repetitive sequences that are present next to spacers [166]. Through a process of CRISPR array transcription, CRISPR RNA (crRNA) and tracer RNA (trRNA) are produced. These two RNAs unite to form guide RNA (gRNA) which interacts with the CRISPR-associated (Cas) protein. It possesses the capability to direct the Cas protein to the target sequence and initiate the necessary cleavage. The cleavage induced by CRISPR is then repaired by DNA repair mechanisms [134]. In the domain of drug designing, a synthetic gRNA is synthesized which is then used along with Cas9 proteins to induce a cleavage on the site of interest. In this way, genetic errors are corrected to cure a diverse set of diseases [66].

### Characteristics of studies and problem distribution

The purpose of this section is to summarise the distribution of AI predictors across 10 different CRISPR tasks. Predictor distribution analysis under individual tasks offers insights into the most active CRISPR tasks. This consolidated distribution provides a centralized platform for researchers to access valuable information about their area of interest.

Table 2 illustrates the distribution of predictors across 10 different CRISPR tasks. Among the 50 predictors, 27 are tailored to predict on/off-target activity in CRISPR [10, 34, 40, 50, 61, 78, 92, 103, 112, 127, 138, 139, 141, 152, 178, 209, 219, 221, 231, 232, 234, 238]. Additionally, approximately 7 predictors are designed to predict acr proteins [35, 47, 57, 63, 109, 193, 242]. Furthermore, 4 predictors are specialized for CRISPR arrays [37, 128, 137, 157] and Cas prediction [145, 147, 187, 235]. Finally, only a few predictors are tailored for tasks such as CRISPR operons [217, 223], gene editing outcomes [6, 105, 116, 163], and aca proteins [216].

Among 10 distinct CRISPR tasks, the prediction of on/off-target activity and acr proteins emerge as prominent trends in current CRISPR research [35, 40, 57, 78, 109, 112, 139, 178, 209, 221, 238, 242]. These tasks garner significant attention due to their crucial roles in refining the specificity and controllability of CRISPR-based genome editing. The ability to accurately anticipate undesired effects prior to laboratory experimentation holds immense value, potentially conserving financial and biochemical resources as well as time. Moreover, integrating predictions of on- and off-target effects and activities with acr proteins holds significant promise for optimizing gene therapies, potentially resulting in safe, inert, and non-detrimental outcomes.

Cas and CRISPR arrays prediction [37, 128, 145, 147, 157, 235] are emerging as prominent research focuses within the realm of CRISPR based gene editing. The ongoing discovery of novel and enhanced Cas systems plays a pivotal role in advancing these tasks, promising better precision and efficacy in gene editing. The identification and characterization of new CRISPR arrays within DNA sequences holds immense potential for optimizing gene editing strategies, facilitating targeted modifications with unprecedented accuracy. While fewer papers are dedicated for the prediction of cleavage, gene editing outcomes, CRISPR operons and acr-associated proteins, their significance cannot be understated. Understanding these processes is essential to fine tune gene editing and to regulate and modulate the activity of CRISPR complexes.

**Table 3:** Benchmark datasets for CRISPR arrays prediction

Datasets	Specie	Type	Positive	Negative
CrisprIdentify [128]	Archea	Train	400	400
		Test	550	200
	Bacteria	Train	600	600
		Test	550	200
CRISPRLstm [37]	-	–	11407	12000

#### CRISPR arrays

**Fig. 6:**
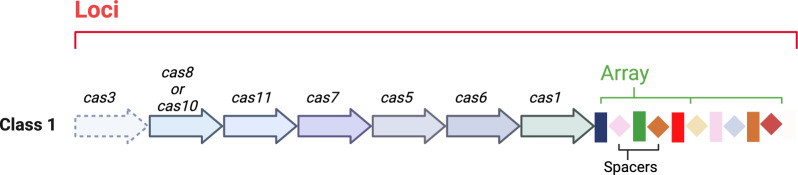
The arrangement of CRISPR loci and CRISPR arrays. (Image created using Biorender.com)

Figure 6 illustrates that CRISPR arrays are short sequences of repetitive DNA (repeats) interspersed with unique sequences (spacers) derived from viral or plasmid DNA that help bacteria to identify external genomes. Once the external genome is identified, CRISPR RNA (crRNA) acts as an immune mechanism, forming a small molecule with Cas proteins that destroys the external genome. Particularly, CRISPR arrays contain two distinct components i.e., spacers, which are small parts of the external genome incorporated inside the bacterial DNA, and repeats, which are small palindromic sequences that are repeated in the CRISPR array. Both of these components help the crRNA to bind with Cas proteins. Research in this field includes the detection of CRISPR arrays and the discrimination between valid and non-valid arrays [37].

Table 3 presents 3 different benchmark datasets developed to identify CRISPR arrays. Mitrofanov et al. [128] gathered archaeal and bacterial CRISPR arrays and generated two different benchmark datasets. On the basis of the these datasets, authors checked the validity of CRISPR arrays. Deshmukh et al. [37] proposed a CRISPR detection method with three stages: detect potential CRISPR arrays, classify repeats, and filter invalid arrays. First, the CRT tool identifies potential arrays from DNA sequences using specific parameters. Next, an LSTM model with a sigmoid activation function scores the repeats. Finally, the method averages these scores to calculate the overall array score, discarding arrays below a certain threshold. The authors assessed the accuracy of classifying short DNA segments as CRISPR repeats using a dataset of 11,407 CRISPR repeats and 12,000 invalid repeats [56, 197]. They validated the CRISPRLstm pipeline with 309 CRISPR arrays from 60 organisms.

#### CRISPR loci

The CRISPR locus consists of the CRISPR array and the Cas genes that form an operon as shown in Fig. 6. The CRISPR locus is responsible for the complete adaptive immune response in prokaryotes, including spacer sensing, crRNA processing, and foreign DNA interference. The arrays store genetic information from previous infections, while the Cas genes encode proteins necessary for processing and fighting invaders.

Nethery et al. [137] created a benchmark dataset for CRISPR loci subtype identification. First, authors downloaded genomes with previously classified CRISPR loci from the National Center for Biotechnology Information (NCBI) [159]. Authors obtained repeats using MinCED [15], retaining all detectable sequences. The data, comprising 7,808 CRISPR loci and 15,669 repeat sequences across 30 subtypes, were used to train the model. Overall, The training set included 12,534 repeats, and the validation set contained 3,135 repeats across 30 subtypes. The dataset can be obtained from the following link https://github.com/CRISPRlab/CRISPRclassify.

Russel et al. [157] created another benchmark dataset for CRISPR loci identification by using MinCED v0.4.2 [15]. They included consensus repeats from all arrays located within 1 kbp of a Cas operon, resulting in a total of 5,838 subtyped repeat sequences. The benchmark dataset can be downloaded from the following link https://github.com/Russel88/CRISPRCasTyper/tree/master.

#### Cas proteins

The CRISPR-associated protein has the purpose of cleaving at the DNA target site. Over the years different Cas proteins have been discovered and designing a CRISPR/Cas system with specific Cas proteins aids in the precise performance of the cleavage [235]. As a result, undesired on-target effects are minimized. A persisting challenge is that the variety and number of available Cas proteins are still not meeting the researchers’ needs, hindering the development of CRISPR/Cas editing tools. The large size of the currently known Cas proteins often leads to limitations in the gene editing process, thereby encouraging the continuous search for smaller Cas proteins. In this field ML and DL techniques contribute to the research by predicting whether a protein has the potential to be a Cas protein or not [235].

Yang et al. [220] proposed the first benchmark dataset for Cas protein prediction dataset. The authors gathered Cas protein sequences from the UniProt database and applied the CD-HIT tool to yield 155 Cas protein sequences. Authors collected non-Cas protein sequences form Uniprot having no or less similarity with Cas protein sequences. This resulted in 155 non-Cas protein sequences. Building on their work, Zhang et al. [235] followed similar protocol to collect Cas protein sequences. In addition, Zhang et al. [235] the non-Cas protein sequences from the work of Yang et al. [220]. Overall, Zhang et al. [235] dataset contained 418 Cas and non-Cas protein sequences.

**Fig. 7:**
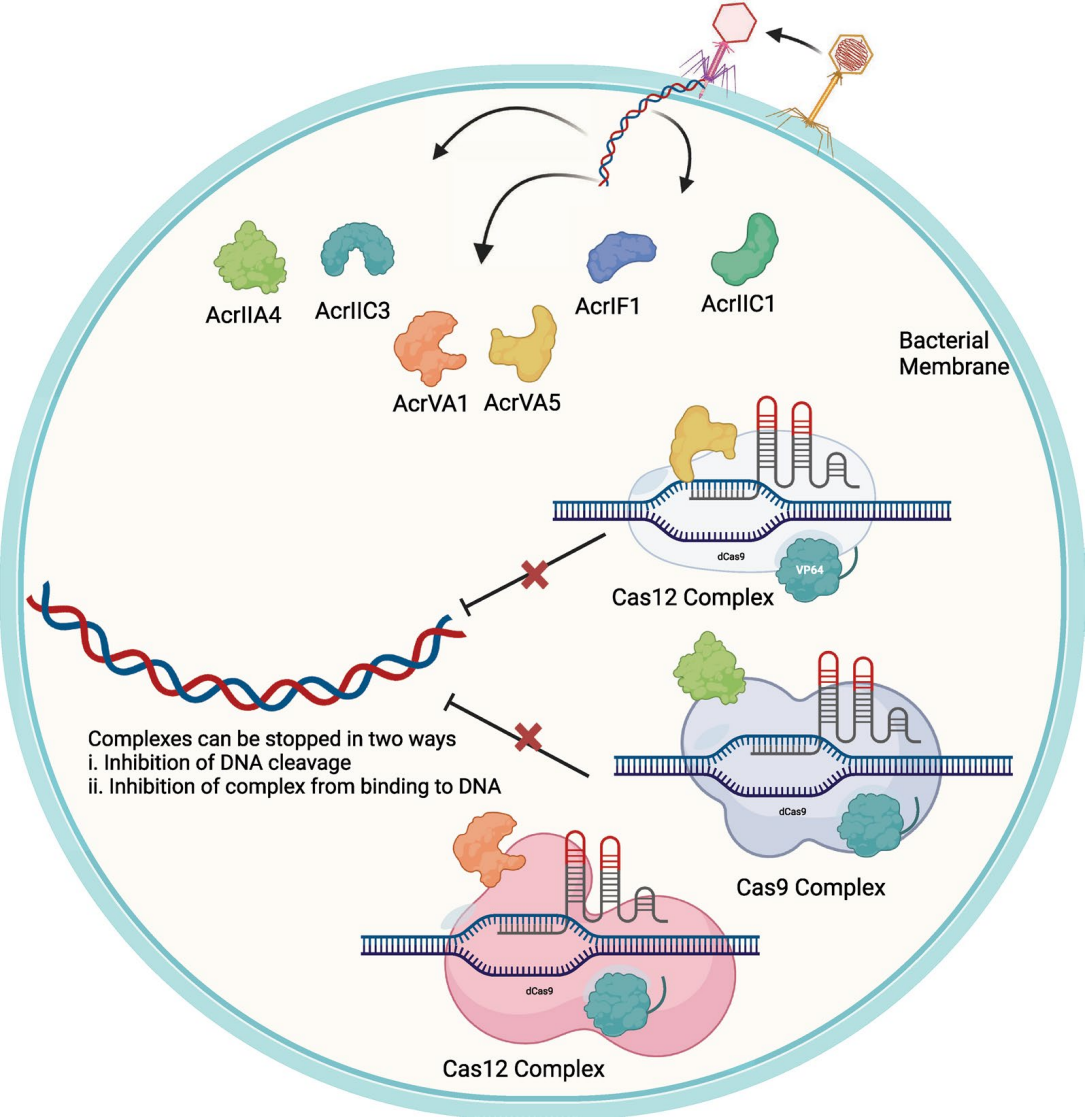
Viral genome contains information about various acr proteins. Once translated, these proteins can interact with Cas complexes and inhibit them from cleaving the viral genome. (Image created using Biorender.com)

#### Anti-CRISPR proteins

Anti-CRISPR (acr) proteins act against the CRISPR mechanism. Figure 7 shows that acr proteins play a crucial role as a control mechanism for the CRISPR system’s activity [225] and can work in two different ways, i.e., they can prevent the Cas-gRNA complex from binding to target DNA, and they can also block cleavage by deactivating the Cas effector [225]. With the help of acr proteins in the gene editing process, timing and precision are enhanced, and undesired effects are mitigated. In terms of acr proteins, there are 3 crucial tasks which include predicting acr family classes [109], binary classification of acr proteins [35], and acr-Cas protein interaction prediction.

**Table 4:** Benchmark Datasets for ACR protein prediction

Data		Positive	Negative	Additional Details	Databases Used	Link	Year
AcrNet-5-fold [108]	Train	1094	1162		paCRISPR, CRISPRDb	https://acranker.pythonanywhere.com/	2023
AcrNet-1 [108]	Train	884	902	From type I-F, II-C, and I-D in anti-CRISPRdb			
	Test	210	260				
AcrNet-2 [108]	Train	904	902	From type I-F, I-E, V-A, I-C, VI-A, VI-B, III-I, III-B, and I-B in anti-CRISPRdb			
	Test	190	260				
AcrNet-3 [108]	Train	962	902	From type I-D, II-C, I-E, V-A, I-C, VI-A, VI-B, III-I, III-B, and I-B in anti-CRISPRdb			
	Test	132	260				
AcRanker [47]	Train	432	432	12 of the proteins are active against subtype I-F CRISPR Cas systems, four against I-E, and four against II-A	AntiCrisprDb	https://academic.oup.com/nar/article/48/9/4698/5819938	2020
	Test		-	–	-	-	-
PreAcrs [242]	Train	412	412		Anti-CRISPRDb, AcrDb, AcrCatalog	https://github.com/Lyn-666/anti_CRISPR/tree/main/data	2022
	Test	176	176				
PaCRISPR [193]	Train	98	902	–	AntiCrisprDb, and literature	https://pacrispr.erc.monash.edu/download.jsp	2020
	Test	26	260				
[57]		488	488	–	AntiCrisprDb, and literature	-	
[35]	Train	205	902	–	-	-	2023
	Test	26	260	–	-	-	

Anti-CRISPRdb [44] categorizes a variety of acr proteins, which inhibit different subtypes of CRISPR systems. For instance, Type I-F [122] includes 12 identified Acr proteins. These proteins inhibit Type I-F CRISPR-Cas systems found in various bacteria such as *Pseudomonas aeruginosa*. Type I-E [226] Acr proteins target the I-E subtype, which is another common type of CRISPR-Cas system. Similarly, Type II-A Acr proteins [230] are used to inhibit Cas9 protein commonly used in gene editing technologies. Type I-C, I-D, III-B, III-I, V-A, VI-A, VI-B, have a varied number of Acr proteins identified that inhibit their respective CRISPR-Cas systems. The sequences of these proteins are updated daily in databases like Anti-CRISPRdb [44], and AcrHub [195]. Based on these databases and types of acr proteins, multiple acr proteins benchmark datasets have been proposed.

Table 4 provides an overview of 6 different benchmark datasets used to train AI acr protein predictors. Li et al. [108] proposed 3 different benchmark acr protein datasets. Authors collected acr proteins from anti-CRISPRdb [44] and PaCRISPR [193]. In order to test the generalizability of the acr protein predictors in a better way, the authors created three different variants of the datasets based on different train and test configurations. For instance, in AcrNet-1, they chose types I-F, II-C, and I-D as testing samples and used the remaining Acrs as training samples. In AcrNet-2, types I-F, I-E, V-A, I-C, VI-A, VI-B, III-I, III-B, and I-B were selected as testing data. In AcrNet-3, types I-D, II-C, I-E, V-A, I-C, VI-A, VI-B, III-I, III-B, and I-B were chosen as testing data.

Etzinger et al. [47] collected acr protein data from the Anti-CRISPRdb [44], ensuring a non-redundant set with a 40% sequence identity threshold using CD-HIT, resulting in 20 verified Acrs for the positive class. This included 12 against subtype I-F, 4 against I-E, 4 four against II-A. They downloaded complete proteomes of source species and filtered out proteins with $\ge$ 40% similarity to known Acrs to form the negative dataset. For independent testing, they used a separate dataset of 20 known Acrs covering various mechanisms and sequences, primarily from the same subtypes as the training set.

Zhu et al., [242] collected 1,378 validated Acrs from Anti-CRISPRdb and 17 new Acrs from NCBI, then used CD-HIT with a 70% identity threshold to filter redundant sequences which resulted in 588 Acrs. These were split into 412 for training and 176 for testing. For negative samples, 1,571 non-Acrs were selected from UniProt based on four strict criteria, 412 were used in training while 176 were used in testing. Finally, training dataset had 412 positive and 412 negative samples, and the test dataset had 176 positive and 176 negative samples.

Wang et al. [193] collected 488 experimentally validated acr proteins from Anti-CRISPRdb and literature. After removing redundant sequences with more than 70% identity, they obtained 98 sequences as positive samples for training. Negative samples were selected based on four criteria: they must not be acrs, must come from phages or bacterial MGEs, must have <40% sequence similarity to each other and the positive samples, and must have lengths between 50 and 350 residues. This resulted in a training dataset of 98 positive and 902 negative samples. For further testing, they collected 26 new acrs with 10% similarity to the training set (except two) and 260 non-acrs using similar criteria, forming an independent dataset with 26 positive and 260 negative samples.

**Table 5:** Benchmark datasets for acr-Cas protein interaction prediction

Type	Positive	Negative	Additional details	Databases	Link	Year
AcrTransAct [63]	132	95	type I-C, I-E, or I-F), and Acr inhibits the CRISPR-Cas system (label 1) or not (label 0)	AcrHub, and antiCRISPRDb	https://github.com/USask-BINFO/AcrTransAct/tree/main/data	2023
AcrCasPPI [132]	107	107	–	PDB, AntiCRISPRDb, and Genbank	https://pypi.org/project/acrcasppi-ml/	2023

Table 5 provides an overview of 2 benchmark datasets for acr-Cas protein interaction prediction. Hasani et al. [63] proposed an acr-mediated CRISPR-Cas inhibition dataset. The dataset comprises 227 pairs of Acr and CRISPR-Cas systems, with 132 pairs showing positive (functional) inhibition and 95 pairs negative (non-functional) inhibition. These sequences are taken from AcrHub [195], Anti-CRISPRdb [44], and several published works. Each sample includes the Acr and Cas protein sequences, organism identity, CRISPR-Cas system type (I-C, I-E, or I-F), bacterial species/strain, and an inhibition label (1 for positive, 0 for negative). Focused on type I CRISPR-Cas systems, the dataset excludes subtypes I-B and I-D due to insufficient information. It features systems from *Pseudomonas aeruginosa*, P*ectobacterium atrosepticum*,* Escherichia coli*, and *Serratia species*.

Murmu et al. [132] developed a Cas-acr interaction dataset: positive (interacting pairs) and negative (non-interacting pairs). They compiled 192 interacting Acr and Cas protein pairs from the Anti-CRISPRDb [44] and removed 85 redundant pairs. Cas protein sequences were retrieved from protein data bank (PDB) [17], UniProt [31], and GenBank [12]. Negative pairs were generated by shuffling amino acid sequences to create a balanced dataset.

#### Off-target activity prediction

**Fig. 8:**
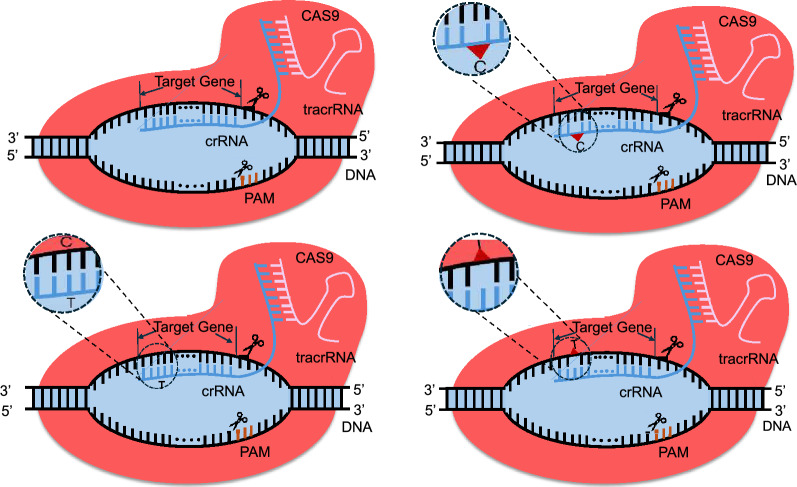
3 different types of off-target effects. A) corresponds to the normal gene editing process. B) refers to the bulge of RNA, C) shows the mismatch case, where the target is not fully recognized and a cleavage is made at the wrong location, and D) a bulge of the DNA

In the CRISPR gene editing process, the single guide RNA (sgRNA) directs the Cas9 protein to the precise location for the intended genetic modification as shown in Fig. 8. This process is not always executed as desired, as Cas9 may cleave at unintended locations. Such unintended cuts can lead to unstable gene sequences and malfunctions in normal genes [21]. This phenomenon is referred to as off-target effects or off-target activity. These effects are influenced by factors such as the structure and length of sgRNA [21]. In this particular task, AI predictors are trained in two different paradigms i.e., classification: Off-target sites are labeled “1” for unintended edits by CRISPR9, while on-target or non off-target sites are labeled “0” for intended edits, regression: a continuous value represents the likelihood or magnitude of off-target activities at the target genomic location.

**Table 6:** Off target activity datasets

Dataset	Set	Positive	Negative	IR	Cell Type(s)	Link	Year
Dhanjal et. al., [39]	Train	6337	7040	1.46	HEK293T K562V U2OS	https://web.iitd.ac.in/crispcut/off-targets/	2018
	Test	2877	4010	–		-	
K562 [29]	-	120	20199	168.32	K562	https://github.com/bm2-lab/DeepCRISPR	2018
HEK239T [29]	-	536	132378	246.97	HEK239T	https://github.com/bm2-lab/DeepCRISPR	2018
CRISPOR [148]	742	408260	550.22	HAP1, HEK293T, K562, and U2OS	$^{a}$	2018	
Zhang et al., [236]	-	26412	26412	1	HEK293T	https://github.com/JiazhiHuLab/CNN_predict	2021
CHANGE-seq [96, 214]		67476	2806151	41.59	Human primary T cells	https://github.com/OrensteinLab/SysEvalOffTarget	2022

$^{a}$ https://academic.oup.com/bioinformatics/article/34/17/i757/5093213

**Table 7:** Off target activity datasets

Type	Technique	Total	Validated Off-targets	Guide RNAs	With Indel	Cell Type(s)	Link
I/1 [182]	CIRCLE-Seq	584949	7371	10	Yes		-
I/2 [115]	GUIDE-Seq	213943	60	6	Yes		https://codeocean.com/capsule/9553651/tree/v1
II/1 [43]	Protein knockout detection	4853	2273	65	No	A375 BV2 HT29	–
II/2 [60]	PCR, Digenome-Seq and HTGTS	10129	354	19	–		–
II/3 [19]	SITE-Seq	217733	3767	9	No	HEK293	–
II/4 [181]	GUIDE-Seq	294534	52	9	No	HEK293T U2OS	–
II/5 [90]	GUIDE-Seq	95829	54	5	No	EGFP U2OS	–
II/6 [115]	GUIDE-Seq	383463	56	22	No	HCT116 HEK293T HL60 Kbm7 K562 U2OS	–

Table 6 and 7 present 14 different benchmark off-target activity prediction datasets that have been developed to train and evaluate AI predictors. Table 6 encompasses 7 different benchmark datasets for off-target activity prediction across six distinct cell types: HEK293T, K562V, U2OS, K562, HAP1, and Human primary T cells. For example, Dhanjal et al. [39] created an off-target activity benchmark dataset using GUIDE-seq [181], SITE-seq [19], and CIRCLE-seq [182]. The inactive targets were chosen from *CRISPCut* [38], resulting in highly imbalanced datasets due to the abundance of negative samples. Chuai et al. [29] developed 2 different datasets for off-target activity prediction using two cell types: 293-related cell lines (18 sgRNAs) and K562 cells (12 sgRNAs). By utilizing *bowtie2* [95], they identified approximately 160,000 potential off-target loci across the genome for 30 sgRNAs, allowing up to six mismatches. This dataset was also highly unbalanced, with roughly 1 in 250 loci identified as off-targets. Zhang et al. [236] proposed a balanced off-target activity dataset of Cas9 variants for HEK293T cell lines. Peng et al. [148] created another dataset from 9 different experiments performed on CRISPR, including Targeted PCR [27, 72, 200], PCR [85, 86], Flanking PCR [153], GUIDE-seq [181], Digenome-seq [85], HTGTS [53], Multiplex Digenome-seq [86], and CIRCLE-seq [182], encompassing a total of 76 gRNAs. Lazzarotto et al. [96] recently introduced a new dataset for off-target activity based on in-vitro and in-cellular experiments i.e., CHANGE-seq (110 gRNAs). In this specific dataset, active on-targets with up to six mismatches were experimentally determined, while inactive off-targets were identified using Cas-OFFinder [9].

Lin et al. [113] classified the off-target effects of CRISPR gRNAs into three categories: a) sites with base mismatches; b) sites with missing bases (RNA bulge or insertion); c) sites with additional bases (DNA bulge or deletion). Instances (b) and (c) are recognized as indel (insertion or deletion) off-target occurrences. Building on the similar idea, 8 different datasets have been proposed which are presented in Table 7. For instance, I/1 [182] and I/2 [115] encompass pairs of gRNA and target DNA sequences exhibiting mismatches and indels. Specifically, I/1 [182] comprises pairs sourced from 10 distinct gRNAs, among which 7371 active off-targets (430 featuring indels) were empirically affirmed through CIRCLE-seq experimentation. Similarly, I/2 [115] comprises pairs sources from 6 different sgRNA with approximately 60 validated active off targets. Furthermore, utilizing the gRNA sequences, Cas-Offinder [9] a flexible tool designed for identifying potential off-target sites of Cas9 RNA-guided endonucleases was employed to acquire inactive off-target sites in the genome associated with the aforementioned two types.

6 independent gRNA-target pairs based off-target activity datasets do not incorporate mismatches and indels together, but rather focus on only mismatches i.e., II/1 $\cdots$ II/6. Donech et al., [43] provided II/1 which contains 65 gRNAs related 4,853 validated off targets with human sequence target CD33, belonging to three different cell lines i.e., A375, BV2, and HT29. Similarly, Haeussler et al. [60] provided II/2 dataset of 19 gRNAs with a total of 350 validated off targets. II/3, proposed by Cameron et al. [19] contains 3,767 positive off-target sites from 9 different gRNAs validated by SITE-Seq. Datasets II/4, II/5, and II/6 comprise validated gRNA-target pairs confirmed through GUIDE-Seq, each sourced from distinct research works: Tasi et al. [181], Listgarten et al. [115], and Kleinstiver et al. [90]. It is noteworthy to mention that Tasi et al. [181], Listgarten et al. [115], and Kleinstiver et al. [90] solely provided the active off-target sites. Consequently, employing Cas-Offinder [9], all potential off-targeting sites with up to six mismatches in the human genome were identified, and the corresponding datasets were formulated.

From the pool of the studies selected for the review in this paper, multiple datasets have been utilized for off-target activity prediction. Störtz et al. [172] and Daneshpajouh et al. [34] utilized the CrisprSQL dataset [171], a comprehensive collection of 17 base-pair resolved off-target cleavage studies on SpCas9, totaling 25,632 samples. It includes data from various cell lines, primarily U2OS, HEK293, and K562.

Toukifuzzaman et al. [178] utilized sgRNA-DNA pairs of DeepCRIPR study [29]. Imani et al. [78] used the K562 and HEK293T cell lines related DeepCRISPR dataset [29] for training DL models. On the other hand, Lin et al. [112] trained and assessed their models on both CRISPOR [60] and GUIDE-seq [181, 214] datasets.

Neu et al. [139] utilized 7 different off-target activity prediction datasets namely, CIRCLE-seq [182] (contains mismatch, insertion, and deletion off-target sites), protein knockout detection (II/1) [43], Digenome PDH (II/2) [60], II/3 SITE [19] and GUIDE-seq I, II, III (II/4, II/5, II/6) [90, 115, 181]. Yang et al. [221] utilized all the datasets presented in Table 7 and K562, HEK293T datasets of Chuai et al. [29]. Toufikuzzaman et al. [178] used the augmented datasets of DeepCrispr [29] with a maximum of six nucleotide mismatches. This specific dataset contains 293-related cell lines (18 sgRNAs) and K562t (12 sgRNAs).

#### On-target activity prediction

When Guide RNA along with CRISPR system is directed for a specific DNA sequence, the Cas9 protein induces double-stranded breaks at that specific genomic location. Subsequently, these breaks are repaired by the cell’s DNA repair mechanisms such as non-homologous end joining (NHEJ) and homology-directed repair (HDR) [94] as shown in Fig. 10. These mechanisms can introduce challenges and potentially cause unwanted effects at the target site, such as insertions and deletions [94]. AI methods are utilized to predict the efficiency of gRNA or on-target activity.

In the era of CRISPR, numerous datasets have emerged to assess on-target activity that stem from various origins such as in vitro experiments, or in vivo studies. This diversity underscores the necessity for novel algorithms to benchmark against these datasets. Considering a similar notion, researchers have recently endeavored to gather disparate on-target activity datasets onto a unified platform. For instance, Haeussler et al., [60] gathered 15 different CRISPR on-target activity datasets. These datasets are subdivided into two main groups on the basis of the origin of the gRNA i..e, from U6 or T7 promoter. Table 8 shows samples statistics of CRISPR on-target activity benchmark datasets based on U6 and T7 promoters. The U6 promoter groups include 12 different datasets which include Wang/Xu HL60 [199, 210], Donech Mouse-EL4 [42], Koike-Yusa 1 M-ESC [91], Chari 293T [22], Donech A375 [43], Hart Repl2Lib1 HCT116 [62], Gandhi Eelectrop. Ciona [55], Farboud C. elegans [51], Ren Drosophilla [155]. Similarly, the T7 promoter based datasets include Varshney Zebrafish [183], Gagnon Zebrafish [54], and Morneo-Mateis Zebrafish [131].

**Table 8:** On-target activity datasets based on U6 and T7 promoters and bacteria

	Dataset	Specie	No. of Samples	Year
U6	Chari 293T	HM	1193	2015
	Doech HS	HM	110	
	Doech MM	MM	150	2014
	Doench azd Hg19	HM	431	2016
	Hart HCT116	HM	4199	2016
	Hart HeLALib1	HM	4217	2016
	Hart HeLALib2	HM	3816	2016
	Hart RPE1	HM	4175	2016
	Xu HL60	HM	2057	2015
	Xu KMB7	HM	2057	2015
	Gandhi	CN	72	2016
	Farboud	CE	50	2015
T7	Gagnon	ZB	111	2014
	Moreno-Mateos	ZB	1020	2015
	Varshney	ZB	102	2015
Bacteria	Guo E.Coli	E.Coli	40, 468	2016

These datasets are available under following repositories https://github.com/maximilianh/crisporPaper, and https://github.com/VKonstantakos/CRISPRedict

**Table 9:** Counts of train and test samples for each CRISPR variant

CRISPR Variant	Train	Test	Link	Year
SpCas9	34713	5415	Clickable Link	2019
SpCas9-NG	.	.		.
VRQR Variant	.	.		.
xCas	.	.		.
Sniper-Cas9	.	.		.
eSpCas9(1.1)	.	.		.
SpCas9-HF.1	.	.		.
HypaCas9	.	.		.
evoCas9	34713	5415		.
HT-Cas9 (kim)	12832	542		2019
Xiang-gRNA	10,592	.		2021

**Table 10:** Samples statistics of protospacer and PAM combinations for on-target activity

Types	Dataset	No. of Samples	Link	Year
LARGE	SpCas9-HF1 (High Fidelity Cas9) (ESP) [190]	58616	https://github.com/izhangcd/DeepHF	2019
	SpCas9-HF1 (High Fidelity Cas9) (HF1) [190]	56887	https://github.com/izhangcd/DeepHF	2019
	WT-SpCas9 (Wild-Type Streptococcus pyogenes Cas9)[190]	55603	https://github.com/izhangcd/DeepHF	2019
MEDIUM	Sniper*	37974		
	SpCas9*	30585		2018
	xCas9*	37738		2018
SMALL	Hart HCT116*	4239	https://github.com/bm2-lab/DeepCRISPR	2015
	HELA (Hart HeLALib1 + HeLALib2)*	8101	https://github.com/bm2-lab/DeepCRISPR	2015
	Wang/Xu HL60*	2076	https://github.com/bm2-lab/DeepCRISPR	2014

Datasets with ’*’ are reported in earlier tables as well

As the CRISPR field is burgeoning, a steady stream of new datasets continues to emerge regarding CRISPR’s on-target activity. For instance, Wang et al. [190] proposed new on-target activity datasets based on the different Cas proteins i.e., SpCas9-HF1, or High Fidelity Cas9 (ESP), which is a modified version of the Cas9 protein derived from Streptococcus pyogenes (SpCas9)and exhibits higher specificity in targeting DNA sequences. Similarly, SpCas9-HF1 (High Fidelity Cas9) (HF1) is another variant of the Cas9 enzyme from Streptococcus pyogenes (SpCas9). Like SpCas9-HF1 (ESP), this version is designed to improve the specificity of CRISPR genome editing. WT-SpCas9 refers to the wild-type form of the Cas9 enzyme isolated from Streptococcus pyogenes. This unmodified version of Cas9 is the original enzyme used in CRISPR genome editing. While it remains a powerful tool for gene editing, WT-SpCas9 may exhibit higher off-target effects compared to engineered high-fidelity variants such as SpCas9-HF1 (ESP) and SpCas9-HF1 (HF1). Researchers often use WT-SpCas9 alongside modified versions to compare their editing efficiency and specificity.

Kim et al., [87, 88, 208] proposed more on-target activity datasets across different settings in CRISPR with the same or enhanced variants of Cas9 protein i.e., SpCas9, SpCas9-NG, VRQR variant, xCas, Sniper-Cas9, eSpCas9(1.1), SpCas9-HF.1, HypaCas9, and evoCas9. The sample statistics are provided in Table 9.

As the influx of datasets continues to grow, researchers endeavor to establish a consensus by proposing various categorizations of datasets. For instance, Zhang et al. [233] devised a taxonomy that classifies on-target activity datasets into 3 groups based on size, i.e., small, large, and medium datasets as shown in Table 10. This approach aids in organizing and understanding the diverse array of datasets available for analysis and research purposes, facilitating more efficient data utilization and fostering collaboration within the scientific community. Although it is simplistic and organized, authors neglect multiple datasets of Kim et al., [88] and the majority of datasets from Table 8 which are initially collected and presented by Haeussler et al., [60].

**Table 11:** On-target activity prediction datasets for epigenome editing

Type	Dataset	gRNA	Genes	Source
CRISPRoff Editing	CRISPRoff-tiling	111682	520	[143]
	CRISPRoff-genome	20221	18779	[143]
	Endogenous Genes (H2B)	326	1	[143]
	Endogenous Genes (CLTA)	415	1	
	Endogenous Genes (RAB11A)	392	1	
	Endogenous Genes (VIM)	528	1	
CRISPRi editing	CRISPRi-activity score	18079	1539	[68]
	hCRISPRi-v2	199523	18549	[68]
	CRISPRi-genome	107595	14361	–
	CRISPRi-K562	111283	520	–
CRISPRa editing	hCRISPRa-v2	198756	18495	–
	CRISPRa-activityscore	2779	236	–

**Fig. 9:**
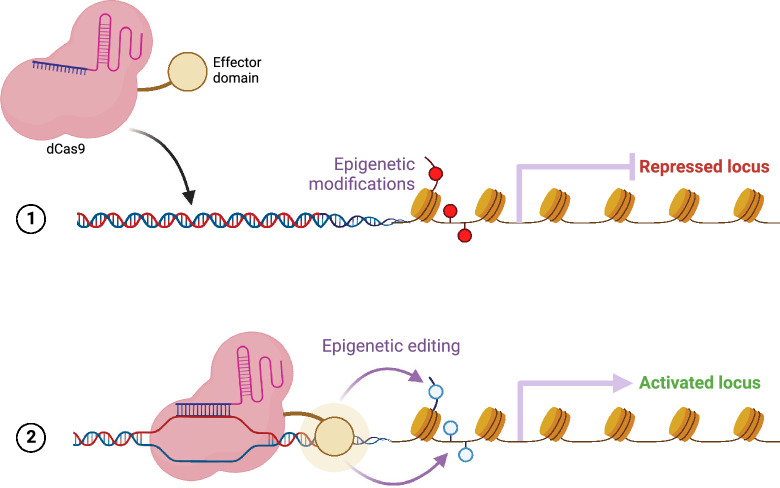
The effects of epigenetic modifications and the potential for reversal using dCas9. The top part shows that epigenetic modifications can cause gene repression. The bottom part demonstrates how dCas9 can be used to perform epigenetic editing to reverse these modifications, leading to gene activation. (Image created using Biorender.com)

Like gRNA’s role in gene editing, it also exhibits activity in epigenome editing as shown in Fig. 9 [219]. This enables the regulation of gene expression without altering the underlying DNA sequence as shown in Fig. 9. Yang et al. [219] gathered these datasets from different literature sources [68, 143]. The statistics of these 9 datasets are provided in Table 11.

**Table 12:** Number of sequences with high and low on-target activities for the four crops. These datasets can be downloaded from http://crispr.hzau.edu.cn/CRISPR-Local/

Crop	Pos. Seq.	Neg. Seq.
Glycine	135,800	122,880
Zea	643,939	442,190
Sorghum	722,906	837,222
Triticum	581,120	429,900

Niu et al. [138] created 4 distinct agronomic species datasets i.e., Glycine max, Zea mays, Sorghum bicolor, and Triticum aestivum by gathering sgRNA sequences with high and low on-target activities from Sun et al. [174] which included experimentally verified seed sgRNAs with known knockout effects. The initial dataset contained around 15,000 sgRNAs from seed experiments. The authors utilized CD-HIT to remove redundant sequences from positive and negative samples. The sample statistics of these datasets are presented in Table 12.

Overall, 27–39 unique CRISPR on-target datasets can be considered to design and benchmark novel on-target activity prediction tools/applications based on the problem setting. Inside each dataset, a sample contains nucleotide PAM sequence along with a numerical on-target activity value.

Over the past three years, 6 different studies have been conducted to enhance the accuracy of on-target activity predictions. Each study utilizes a specific set of datasets to train and evaluate the performance of ML and DL models. For instance, Xiao et al. [209] trained and assessed the performance of their DL model namely, AttCRISPR on 3 different publicly available datasets from DeepHF [111] namely, WT-SpCas9, eSpCas9(1.1) and SpCas9-HF1. These datasets contain 55604 (WT-SpCas9), 58617 (eSpCas9(1.1)), and 56888 (SpCas9-HF1) sgRNAs with continuous activity values.

Dimauro et al. [40] utilized the datasets gathered by Xu et al. [213] which are also presented in Table 8. Particularly, the authors used 10 out of 15 different datasets namely, Wang/Xu HL60 [199, 210], Donech Mouse-EL4 [42], Chari 293T [22], Donech A375 [43], Hart Repl2Lib1 HCT116 [62], Gandhi Eelectrop. Ciona [55], Farboud C. elegans [51], Varshney Zebrafish [183], Gagnon Zebrafish [54], and Morneo-Mateis Zebrafish [131]. Similarly, Zhang et al. [231], Rafid et al. [152], Li et al. [103], and Fanaras et al. [50] utilized only 4 different datasets namely, Hart Repl2Lib1 HCT116 [62], Chari 293T [22], Hart Repl2Lib1 HCT116 [62], and Wang/Xu HL60 [199, 210]. It is important to mention that Zhang et al. [231], Li et al. [103], and Fanaras et al. [50] did not utilize the original versions of these datasets. Instead they made use of augmented datasets as done in DeepCrispr study [29]. The researchers expanded these datasets by introducing two mismatches in the PAM-distal region of original sgRNA sequences, a technique that does not affect cleavage efficacy. This process generated approximately 200,000 unique sgRNAs, each assigned the same efficacy labels as the original sequences. The augmented dataset provides a diverse and biologically meaningful set of sgRNAs for training purposes.

Previous studies have shown that PAM-distal region has a high tolerance for sequence mismatches (Kim et al., 2016; Kleinstiver et al., 2016). To be specific, gRNAs with two mismatches in the first two positions from the 5’ end has little influence on cleavage efficiency (Doench et al., 2014; Doench et al., 2016). Inspired by these studies, Chuai et al. applied a data augmentation procedure by changing each gRNA into a new one with two mismatches in the PAM distal region (Chuai et al., 2018). Consequently, a 23-nt gRNA sequence can be expanded into 16 gRNAs with identical cleavage efficacy. The augmented dataset was generated from  15,000 gRNAs with known on-target cleavage efficacy. By adopting this data augmentation strategy, they obtained 180,512 non-redundant gRNAs. Each observation in the data contains a 23-nt gRNA sequence and its corresponding cleavage efficiency. In this work, we used this augmented dataset as the benchmark data for model selection and pre-training.

Ham et al. [61] created a new on-target activity prediction dataset recently with a motivation that current models poorly predict SpCas9/sgRNA activity because the underlying datasets are inaccurate and fail to distinguish between cleavage activity and toxicity. To address this, authors utilized a two-plasmid positive selection system to generate high-quality data that accurately measures SpCas9/sgRNA cleavage activity and separates it from toxicity. It is important to mention that the last study related to on-target activity prediction explores the performance of DL predictors on 9 different datasets that are presented in Table 10 and discussed earlier [233]

Noshay et al. [141] utilized spCas9 dataset as presented in Table 9. In addition, Konstantakos et al. [92] provided a web server of existing tools by training and assessing these models on gRNAs expressed in U6 and T7 promoters. U6 and T7 promoter based datasets are already discussed earlier and presented in Table 8.

**Table 13:** Sample statistics of benchmark datasets for gene editing outcome prediction

Name	Cell Line	Indels	Deletions	Samples	Link	Year
Apindel [116]	-	-	-	-	https://github.com/MoonLBH/Apindel	2022
Lindel [23]	HEK293T	21	536		https://github.com/shendurelab/Lindel/tree/data_analysis	2019
SPROUT [101]	T cell	9 types statistics of the repair outcomes such as average insertion length.		1603	-	2019
FORECasT [6]	K562, RPE1, iPSC, CHO, HAP1, mESCs	20	420	31617	https://elixir.ut.ee/forecast/	2019
InDelphi [163]	HEK293, K562, HCT116, mESCs, U2OS	4	149		https://github.com/maxwshen/indelphi-dataprocessinganalysis	2018

#### CRISPR gene editing outcome prediction

**Fig. 10:**
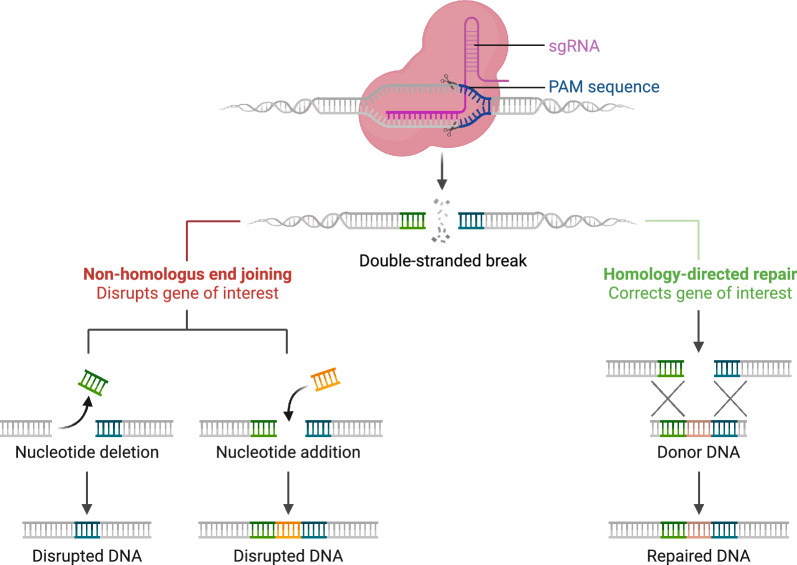
DNA cleavage is repaired by two different pathways i.e., NHEJ and HDJ. NHEJ repairs DNA breaks by directly joining the broken ends, often resulting in small insertions or deletions. HDR uses a homologous sequence as a template to accurately repair DNA breaks which ensures high-fidelity restoration. (Image created using Biorender.com)

Upon locating the target site and inducing a double-stranded break, the cell’s DNA repair mechanisms are activated (Fig. 10). These repair mechanisms include i.e., Homology-directed repair (HDR), and end joining. In a HDR, cells repair the damage by copying from the sister chromatid, filling in gaps around the break site. Scientists can exploit this by introducing a DNA template similar to the CRISPR cut’s surroundings but with a modification. The cell uses this template for repair, resulting in precise, controllable DNA editing at the target location. In addition, the repairs done by end joining i.e., microhomology-mediated end-joining (MMEJ) and non-homologous end-joining (NHEJ), are not controllable as compared to HDR. End joining can be seen as a haphazard attempt to repair the cut in a way that prevents CRISPR from targeting it again. This process results in diverse and heterogeneous insertions and deletions across different cells. While HDR has been the preferred method in genome editing, end joining is often considered undesirable noise, despite being more efficient than HDR Table 13.

Upon completion of the repair process, various disease-related mutations may occur, including insertions, deletions, frameshifts, inversions, translocations, and point mutations. This field encompasses predicting gene editing outcomes [105]. This specific task includes multi-class classification with soft labels. For instance, the types of mutations are predicted (MH deletions, MH-less deletions, and 1 bp insertions) with the likelihood of specific mutation.

A handful number of approximately 5 tools for gene editing outcome prediction tools have been developed till now, out of which 3 proposed novel benchmark datasets. For instance, Shen et al. [163] proposed the very first gene editing outcome prediction tool namely, InDelphi. InDelphi managed to predict 90 classes of MH Deletion, 59 classes of Non-MH Deletion, and 4 classes of 1 bp Insertion. Authors created a benchmark dataset of 1,095 target sites from mouse and human cells i.e., HEK293, K562, HCT116, mESCs, and U2OS [163]. Similarly, ForeCast generated candidate mutations for each gRNA in synthetic contructs to predict repair outcomes [6]. Overall, it had approximately 440 mutational outcomes and more than 31 thousand samples. SPROUT [101] predicts various statistics related to gene editing outcomes such as the fraction of mutant reads with an insertion/deletion, fraction of total reads with insertion/deletion, average insertion length given an insertion, average deletion length given a deletion, diversity, most likely inserted base pair and finally the edit (mutation) efficiency of the CRISPR outcome [101]. Using convolutional neural networks (CNNs) and neural architecture search (NAS), CROTON [105] automates the prediction of 1 bp insertion and deletion probabilities, as well as deletion and frameshift frequencies, directly from raw sequences without any prior knowledge. CROTON [105] utilized the datasets of ForeCast and SPROUT, where the models were trained on synethic construct dataset from ForeCast and evaluated on endogenous T-cell dataset from SPROUT. Apindel [116] uses ForeCast and Lindel datasets and predicts 557 different labels related to different mutations such as 1bp insertion $\cdots$ $\ge$ 3bp insertions, and 5 kinds of 1bp insertions, 6 kinds of 2bp insertions, $\cdots$ 32 different kinds of 29bp insertions.

#### Acr-associated proteins

The acr-associated (aca) protein can be described as a defense mechanism of the bacterial cell against acr proteins [216]. They hinder the acr-protein from blocking the cleavage of the Cas protein. Therefore aca-proteins can be seen and used as a regulatory mechanism for CRISPR gene editing. In this field, researchers try to predict aca proteins and their associated acr-aca operons [216].

While the genomic locations of acr proteins are diverse, they often coexist near the gene loci of aca-proteins. Their genes oftentimes form an operon with the genes encoding for acr proteins [100]. An operon is a functional unit within the genomic DNA. Identifying these operons contributes to the improvement of gene editing tools [217].

Although Yang et al. [216] proposed a framework for the identification of aca proteins and their operons, there is no evidence suggesting the application of AI in this domain or any relevant benchmark datasets [217].

#### Other tasks

While tasks like on-target and off-target effects, anti-CRISPR (Acr) proteins, and CRISPR arrays have garnered significant attention, there are other topics within the CRISPR research landscape that remain less explored. These tasks include CRISPR-Cas system identification, and crRNA classification. These topics have been covered in detailed earlier, therefore, hereby we only discuss their relevant datasets.

[145] constructed CRISPR-Cas systems datasets by collecting Cas protein sequences from classified archaeal and bacterial CRISPR-Cas systems available in public databases such as NCBI. The sequences were clustered using the Markov Cluster Algorithm to identify protein families, and Hidden Markov Model profiles were used to determine the presence of specific proteins within CRISPR-Cas systems. The final dataset consisted of thousands of samples categorized into 17 distinct CRISPR-Cas subtypes, providing a robust foundation for training and evaluating the machine learning models.

[147] developed crRNA classification dataset by using CRISPR-Cas systems from the CRISPRCasdb [149] database, with CRISPR arrays labeled by their co-localized Cas system types. The dataset included multiple major classes, each with over 1,000 samples.

**Table 14:** A pool of CRISPR related databases

	Database	Data Type	URL	Description
CRISPR Arrays	CRISPRBank [14]	Arrays, repeats, and spacers in FASTA	Link	CRISPRBank contains analysis of genome from RefSeq 95 July, 2019. Particulalry, CRISPRDetect 2.4 was employed to comprehensively analyze all 151,845 bacterial genomes and 855 archaeal genomes. In total, there are 132,379 CRISPR arrays and 1,992,510 spacers.
	CRISPRCasDb [149]	FASTA	Link	CRISPRCasdb contains comprehensive data on CRISPR-Cas systems including information on 2,086 CRISPR arrays and 130,293 spacers, along with details on 19,232 Cas proteins and 7,125 associated Cas proteins.
Acr	AcrHub [195]	XLSX, FASTA	Link	AcrHub offers extensive annotations and functional data for anti-CRISPR associated proteins. It features information on 1,800 proteins and their interactions, spanning various species within the bacterial and archaeal domains.
	Anti-Crisprdb [75]	XLSX, CSV, JSON	Link	Anti-CRISPRdb catalogs a wide array of anti-CRISPR proteins with detailed annotations, encompassing sequences and structural data for 1,200 proteins. The database covers anti-CRISPR proteins found in numerous bacterial species, providing insights into their diversity and functionalities.
	AcrDb			AcrDB is a comprehensive database providing sequences and structural information of anti-CRISPR proteins. It includes data on 2,500 anti-CRISPR proteins across diverse bacterial and archaeal species.
	AcrCatalog [58]	FASTA	Link	AcrCatalog is a specialized database that catalogs anti-CRISPR proteins and their interactions across various CRISPR-Cas systems. It contains sequences, structural information, and functional annotations for approximately 16,919 putative acr proteins. These proteins are associated with specific CRISPR-Cas systems, including Cas-IA to IE, Cas-IIA to IIC, Cas-IIIA to IIID, Cas-IVA, Cas-VA, and Cas-VIA to VIC.
Aca	UniProt Database [31]	TXT, FASTA, XML, JSON	Link	Universal Protein Resource database is a resource for protein sequence and annotation data
	AcrCatalog [58]	TXT	Link	Anti-CRISPR proteins predicted with ML [59]
	AcrHub [195]	XLSX, FASTA	Link	AcrHub predicts Anti-CRISPR proteins
Cas	CasPDB [175]	FASTA	Link	CasPDB is an integrated database housing 287 reviewed Cas proteins, 257,745 putative Cas proteins, and 3,593 Cas operons from 32,023 bacterial species and 1,802 archaeal species. The database comprehensively contains all 3,593 putative Cas operons, including 328 operons associated with the type II CRISPR-Cas system.
	CRISPRCasdb [149]	SQL, FASTA	Link	CRISPRCasdb contains CRISPR arrays andcas genes from complete genome sequences
	UniProt Database [31]	TXT, FASTA, XML, JSON	Link	Universal Protein Resource database is a resource for protein sequence and annotation data
	CasPedia [2]	FASTA	Link	CasPedia is an annotated database for Cas proteins from bacteria and archaea, featuring 287 reviewed Cas proteins, 257,745 putative Cas proteins, and 3,593 Cas operons from 32,023 bacterial and 1,802 archaeal species. It offers free access, a user-friendly interface, and details on all operons, including 328 from the type II CRISPR-Cas system.
On/Off target activity	cripsrSQL	CSV	Link	crisprSQL is a SQL-based database forCRISPR/Cas9 off-target cleavage assays and epigenetically annotated, base-pair resolved cleavage frequency distributions
	Ensembl BioMart	FASTA, GTF,GFF, SQL	Link	Ensembl is a genome browser for vertebrate genomes that supports research in comparative genomics, evolution, sequence variation and transcriptional regulation,BioMart is a data mining tool
	crisprSQL	CSV	Link	crisprSQL is a SQL-based database forCRISPR/Cas9 off-target cleavage assays and epigenetically annotated, base-pair resolved cleavage frequency distributions
	CRISPOR	TSV	Link	Data from CRISPOR paper [60]

#### CRISPR databases for the development of new benchmark datasets

This section provides an overview of databases that can be used to develop novel CRISPR-related benchmark datasets. Additionally, it entails the types and quantities of data available in 17 different databases which can help researchers to identify valuable resources for compiling comprehensive and diverse datasets necessary for effective CRISPR research.

The rapid advancements in CRISPR technology have generated a vast amount of data, leading to the creation of numerous databases. Table 14 summarizes the list of public databases categorized based on different CRISPR tasks. These databases encompass data related to various aspects of CRISPR systems, such as CRISPR arrays, acr proteins, operons, Cas proteins, and on/off-target activities. This abundance of data presents a significant opportunity for the development of novel benchmark datasets, which can enhance the performance and accuracy of AI tools designed for CRISPR research.

Databases such as CRISPRBank [14], and CRISPRCasDb [149] provide a wealth of sequences and annotations for CRISPR arrays. These databases include data on hundreds of thousands of CRISPR arrays and spacers from a vast number of bacterial and archaeal genomes. This creates opportunities for training AI predictors to accurately identify and annotate CRISPR arrays in newly sequenced genomes.

Databases such as AcrHub [195], Anti-Crisprdb [44], AcrDb [75], and AcrCatalog [58] offer extensive data on anti-CRISPR proteins, including their sequences, structures, and functional annotations. These datasets span a wide variety of species which provides a comprehensive view of acr proteins diversity. The richness of this data can be utilized to create benchmark datasets for training AI predictors that predict acr proteins based on sequence data. Moreover, these datasets can be used to develop models to map and predict interactions between acr proteins and CRISPR-Cas systems, as well as benchmark tools that annotate the function and efficacy of acr proteins.

The CasPDB [175], CRISPRCasdb [149], UniProt [31], and CasPedia [2] contain extensive data on Cas proteins, including reviewed and putative proteins, as well as comprehensive operon information. This presents opportunities for using these datasets to benchmark AI tools that predict the three-dimensional structures of Cas proteins. Additionally, benchmark datasets can be developed for annotating the function of Cas proteins in various CRISPR-Cas systems, and tools can be created to study the evolutionary relationships between different Cas proteins and operons.

Databases such as crisprSQL [171], Ensembl BioMart [222] provide valuable data on the on/off-target activity of CRISPR-Cas systems. These datasets include detailed information on off-target cleavage assays and epigenetically annotated cleavage frequency distributions. They offer opportunities to benchmark AI predictors that predict off-target effects of CRISPR-Cas editing and develop datasets that help optimize CRISPR tools for higher specificity and reduced off-target activity. Additionally, these datasets can be used to create benchmarks for assessing the safety and efficacy of CRISPR-based gene editing in various organisms.

In conclusion, the diverse and extensive CRISPR-related databases provide a rich source of data for the development of novel benchmark datasets. These datasets can significantly advance AI tools in CRISPR research, improving the understanding and application of CRISPR technology. By leveraging these opportunities, researchers can enhance the accuracy, functionality, and safety of CRISPR-based applications, driving forward the fields of genetics and biotechnology.

**Table 15:** Features and feature engineering methods used in the reviewed papers

	Study	Feature Engineering
On/Off target activity	[221, 232]	K-mer embedding (default embedding layer of keras or Pytorch)
	[141]	GC content, temperature of melting of the DNA duplex, minimum free energy (calculated with ViennaRNA), distance of the target sequence to the closest downstream PAM, location relative to the target gene, HOMO-LUMO energy gap.
	[78]	W2vec embeddings
	[178]	OHE
	[92]	nucleotide composition, position-specific nucleotides, GC content, number of Adenine in the middle, presence of certain motifs
	[127]	Position-independent nucleotides, position-independent dinucleotides, position-specific nucleotides, position-specific dinucleotides, GC content, melting temperature, self-folding energy, Shannon entropy
	[234]	Basic Sequence features/aligned sequence features of gRNAs and targets, mismatch positions, PAM nucleotides in the target sequences, numbers of mismatches
	[219]	One hot encoding, nucleosome positioning features, chromatin accessibility, DNA methylation, gene expression
	[50, 103, 231]	One hot encoding of gRNA and epigenetic information including CTCF binding, H3K4me3, chromatin accessibility, and DNA methylation.
	[209]	Embedding and one hot encoding
	[152]	sequence-based features, position-independent features, position-specific features, n-gaped di-nucleotides
	[139, 187]	One hot encoding
	[172]	Guide and target loci, sequence, cell line, assay type cleavage frequency, CRISPRoff score, nucleosome organisation-related features/scores such as GC content, Nucleotide BDM, NuPoP Affinity
	[228]	GC content, frequency of k-mers, number of poly-T segments, length of the longest poly-T segment, position-dependent k-mer (k=1, 2) instances, melting temperatures, minimum free energy metrics
Acr proteins/Activity	[63]	Pre-trained Evolutionary Scale Modeling (ESM) protein transformer, NetSurfP-3.0 based secondary structure features
	[35]	Dipeptide composition (DPC), Composition-transition-distribution (CTD), Position specific scoring matrices, PSSM-composition, DPC-PSSM, PSSM-AC, RPSSM
	[109]	RaptorX based structure and solvent accessibility features , Transformer embeddings from ESM-1b, POSSUM, and one hot encoding
	[242]	Sequence: Amino acid composition (AAC), Pseudo amino acid composition (PAAC), Composition of k-spaced amino acid pair (CKSAAP), dipeptide deviation from expected mean (DDE), dipeptide composition (DPC) Evolutionary: PSSM-composition, DPC-PSSM, PSSM-AC, RPSSM, PSSM-SMITH. Pretrained: LM, SSA, TAPE-Bert, Unirep, W2vec, ESM, ProtTrans
	[57]	Containing Genome is Self-Targeting, Directon Annotated Protein Fraction, Directon Protein Lengths Mean, Directon Size, Protein has HTH-Downstream, Protein Length, Protein Hydrophobicity
	[193]	PSSM-composition, DPC-PSSM, PSSM-AC, and RPSSM
	[47]	Conjoint-Triad
Edit. Out.	[105]	1 bp insertion frequency, 1 bp deletion frequency, deletion frequency, 1 bp frameshift frequency, 2 bp frameshift frequency, total frameshift frequency
	[163]	MH length MH GC frac. Del. length, Del. length
	[6]	-
	[101]	One hot encoding and genomic features
	[116]	GloVe and positional embeddings
C-arrays	[128]	Repeat length, number repeats, repeat similarity, AT richness, average spacer length, spacer similarity, repeat number mismatches, spacer evenness, MFE score, ORF score, tandem protein score, BLAST score known repeats, BLAST score similarly known repeats
	[37]	Randomly initialized Embeddings
C-loci	[157]	k-mer counts (4-mer)
	[137]	Length, GC content, palindromic index, k-mers
Cas proteins	[220]	Di-peptide composition
	[235]	Amino Acid Composition (AAC), Adaptive skip dipeptide composition (ASDC), Composition of K-Spaced Amino Acid Pairs (CKSAAP), Dipeptide Deviation from Expected Mean (DDE), Quasi-Sequence Order (QSO), Dipeptide Composition, DPC.

### Feature extraction methods in AI driven CRISPR tasks

This section provides an overview of the most commonly utilized feature extraction methods. First, we categorize these feature extraction methods into groups and then explore their distribution across 10 different CRISPR tasks.

Table 15 enlists 75 unique feature extraction methods and their utilization in 10 different CRISPR tasks. These feature extraction methods can be categorized into several groups. For instance, sequence-based feature extraction methods include methods like k-mer [157], nucleotide composition [92], dipeptide composition [242], position-specific nucleotides [92], one hot encoding [209], and position-specific scoring matrices [35]. Structural and physicochemical properties-based methods include GC content [137], melting temperature [127], minimum free energy [228], NetSurfP$-$3.0 [63] and RaptorX-based [109] secondary structure features, and deletion frequencies [105]. Epigenetic and genomic features-based methods encompass nucleosome positioning [219], chromatin accessibility [219], DNA methylation [219], gene expression, and other epigenetic information. Embeddings-based methods include methods such as GloVe [116], positional embeddings [116], and transformer embeddings from models like ESM-1b [63]. Lastly, miscellaneous methods comprise Shannon entropy [127], cell line characteristics [172], CRISPRoff scores [172], ORF scores [128], tandem protein scores [128], and various repeat and spacer-related representation [128].

A deeper analysis of Table 15 reveals that certain feature extraction methods are important in specific CRISPR-tasks because of their ability to capture critical biological information. For on/off-target activity, sequence-based methods such as k-mer, nucleotide composition, and position-specific nucleotides are frequently utilized because they provide detailed insights into the sequence-specific interactions and potential mismatches [50, 103, 209, 219, 228, 231]. Epigenetic and genomic features, including nucleosome positioning, chromatin accessibility, and DNA methylation, are also crucial as they offer context about genomic accessibility and regulation, influencing CRISPR efficacy [103, 219]. It is important to mention here that one hot encoding proves to be quite effective in representing gRNA and DNA sequences for on/off-target activity prediction [50, 103, 209, 228]. This happens because one hot encoding ensures that all possible sequence variations and mismatches are explicitly represented. This allows ML/DL to accurately learn and differentiate the subtle sequence patterns that influence CRISPR targeting efficacy and off-target effects. By retaining the full granularity of sequence data, one hot encoding helps models identify critical nucleotide positions and motifs that are essential for high-fidelity CRISPR targeting. This precision is particularly important given the potential consequences of off-target effects in CRISPR tasks, making one hot encoding a reliable and effective method for on/off-target activity prediction [50, 103, 209, 228].

In acr proteins and their activity prediction, protein-based features like pre-trained models (e.g., ESM), secondary structure features, and dipeptide compositions are vital for understanding protein structure and function, which is key for predicting acr proteins and their activity. Evolutionary features such as position-specific scoring matrices provide insights into conserved sequences and structural stability, enhancing Acr protein prediction accuracy. For gene editing outcomes prediction, structural and physicochemical properties like insertion/deletion frequencies and MH length/GC fraction are essential because they directly influence the types and frequencies of CRISPR edits. Embeddings and learned representations, such as GloVe and positional embeddings, capture contextual and positional information, improving editing outcome predictions. In CRISPR array prediction, sequence-based and miscellaneous features like repeat length, spacer similarity, and AT richness are important as they are specific to the structure and composition of CRISPR arrays, aiding in their identification and classification. For CRISPR Loci, sequence-based and structural features like k-mer counts and palindromic index help characterize the loci where CRISPR systems are integrated, facilitating their identification. In Cas Proteins, protein-based features such as di-peptide composition, amino acid composition, and advanced methods like adaptive skip dipeptide composition (ASDC) and quasi-sequence order (QSO) are crucial for capturing detailed information about protein sequences and structures, essential for predicting Cas protein functions. Overall, sequence-based features and protein-based features are fundamental across multiple tasks, while epigenetic and genomic features are vital for on/off target activity, and structural properties are crucial for acr protein prediction, demonstrating the importance of capturing diverse biological patterns for accurate CRISPR-related predictions.

Though the mentioned and general DL approaches gain more and more popularity, the feature extraction procedure remains a black box for researchers using these approaches in genomics, leading to a lack of interpretability of models [167]. But to truly understand the aspects of CRISPR, interpretability is crucial. Handcrafted features provide a way to interpret and analyze models so that certain aspects of CRISPR can be derived from experiments. Unfortunately, the creation of handcrafted features frequently is a time-consuming and complex task, where many decisions have to be made that influence models’ potential outcomes and performance in many ways.

### Classifiers and regressors utilization in AI-driven CRISPR tasks

This section presents insights into distinct types of classifiers and regressors that have been utilized to develop AI-driven applications for 10 distinct CRISPR tasks. It thoroughly examines emerging trends of classifiers and regressors across distinct CRISPR tasks.

Table 18 provides an overview of 35 classifiers/regressors that are used to develop AI-driven applications for 10 distinct CRISPR tasks. These classifiers/regressors include: convolutional neural networks (CNNs) [172], recurrent neural networks (RNNs) [178], long short-term memory networks (LSTM) [78], fully convolutional networks (FCN) [109], bidirectional long short-term memory networks (BiLSTM) [221], neural architecture search for CNNs (NAS-CNN) [105], bidirectional gated recurrent units (BiGRU) [172], gated recurrent units (GRU) [78], bidirectional encoder representations from transformers (BERT) [117], hierarchical neural networks (HNN) [221], knowledge-infused neural networks (KINN) [238], attention-based CNN [232], multilayer perceptrons (MLP) [209], support vector machines (SVM) [193], k-nearest neighbors (KNN) [147], logistic regression (LR) [92], random forests (RF) [92], extreme gradient boosting (XGBoost) [92], light gradient boosting machine (LightGBM) [242], categorical boosting (CatBoost) [242], extra trees [128], hidden Markov models (HMM) [157], gradient boosting decision trees (GBDT) [235], classification and regression trees (CART) [145], elastic net logistic regression (ENLOR) [127], iterative random forests (iRF) [141], random intersection trees (RIT) [141], and reinforcement learning in the form of collaborative multi-agent reinforcement learning (CMT-MARL) [10].

**Fig. 11:**
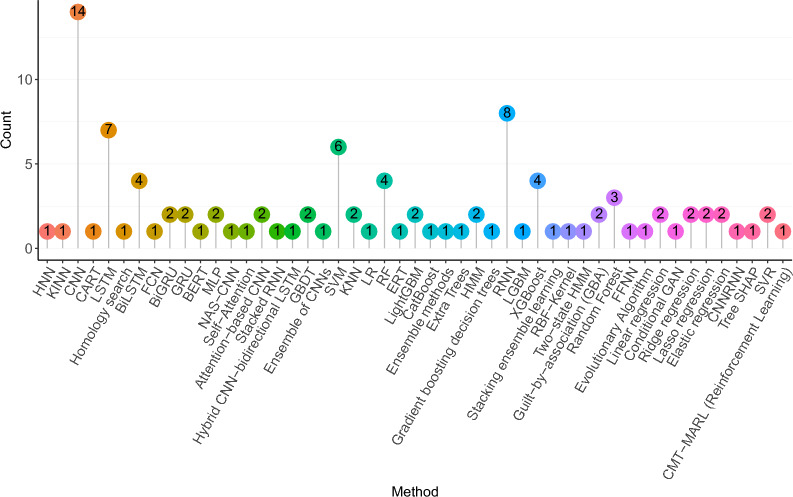
Overall count of classification/regression methods used to develop CRISPR-related applications

These methods can be broadly classified into three different categories i.e., ML, DL, and generic which includes methods from statistics, and reinforcement learning (RL). A deeper analysis of Table 18 and Fig. 11 reveals that DL-based methods have been utilized the most among the 3 categories. Particularly, CNNs, RNNs, LSTMs, and GRUs have been used commonly. The prime focus of researchers has been on CNNs because of multiple reasons. For instance, CNNs are highly effective in capturing spatial hierarchies in data due to their convolutional layers, making them suitable for various CRISPR-related tasks such as predicting off-target and on-target activities [24]. This is crucial in genome editing applications where understanding and minimizing off-target effects are essential for ensuring precision and safety. In addition to CNNs, methods such as RNNs and their variants (like LSTMs and GRUs) are frequently employed due to their ability to handle sequential data, which is valuable in tasks involving time series or order-dependent biological data such as gene editing outcomes or CRISPR array predictions [48]. Moreover, in some studies, researchers have harnessed the potential of CNNs to extract global features from sequence data and combined this with the contextual learning capabilities of RNNs, LSTMs, and GRUs [187]. This powerful combination leverages the strengths of both architectures: CNNs excel at identifying spatial patterns and features across the entire sequence, while RNNs, LSTMs, and GRUs are adept at capturing temporal dependencies and sequential relationships. Integration of distinct architectures into one predictor enables researchers to develop more sophisticated predictive pipelines capable of providing more. Despite the advantages of DL methods, they also suffer due to some limitations. First, DL models require an extensive amount of data to train their weights [98]. In addition, a thorough hyperparameter tuning is required to obtain suitable results. Particularly, if training data is not representative of the broader sample population, DL methods can inherit and amplify biases present in the data which leads to faulty and inaccurate predictions [140]. Finally, DL methods are black box, which means that it is challenging to interpret how DL models make their predictions which can be challenging in sequence analysis tasks [186].

Figure 11 reveals that among ML models, SVM, RF, and XGBoost have been commonly utilized [35, 57, 242]. These models typically perform well under scenarios where features are hand-crafted. Hand-crafted features, derived from domain-specific knowledge, can significantly enhance the performance of ML models by providing relevant and discriminative information [133]. In CRISPR-related studies, carefully designed features that capture the biological intricacies of genomic sequences, protein interactions, and gene editing outcomes can lead to more accurate and reliable predictions. SVMs, with their ability to find optimal decision boundaries, and XGBoost, with its powerful boosting framework, are particularly effective in leveraging these features to achieve high prediction performance. Similar to DL methods, ML methods also have certain limitations. For instance, hand-crafted features are first complex to generate and can increase the dimensionality significantly [25]. Due to this, such models become data sensitive and they fail on noisy samples.

**Fig. 12:**
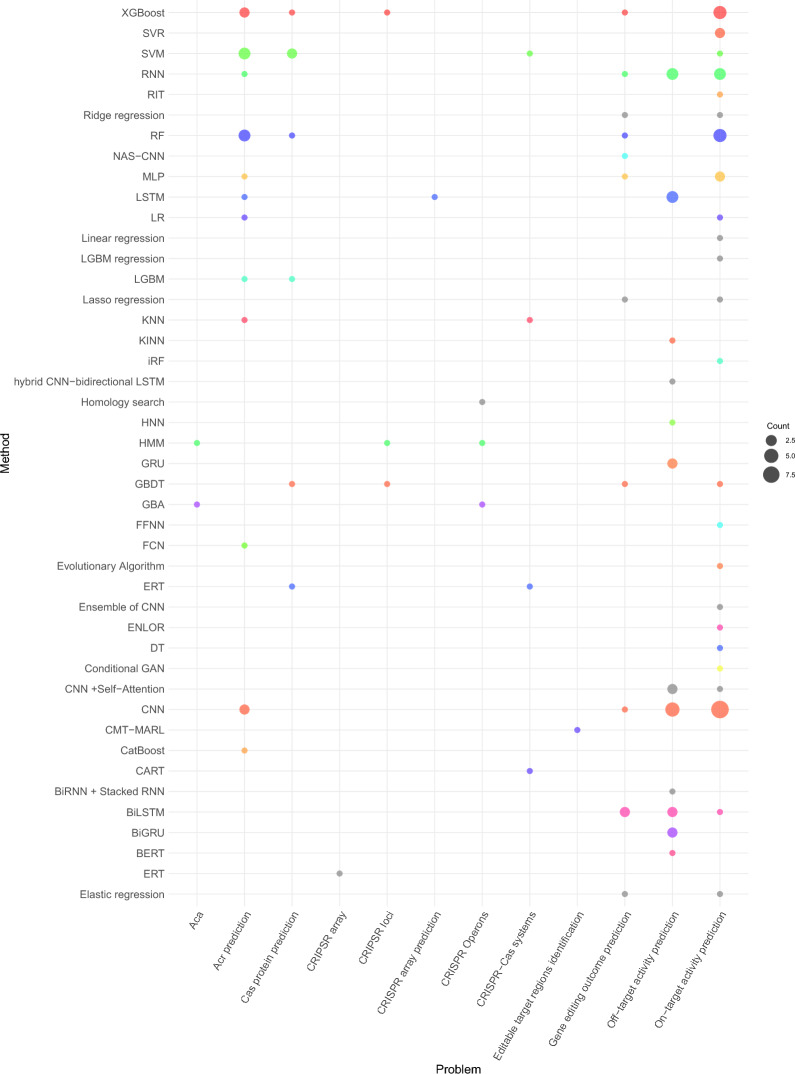
Distribution of AI predictors across different CIRSPR tasks

Figure 12 illustrates the distribution of AI predictors across various CRISPR tasks. Two distinct patterns emerge from this data: first, the specific methods that have been utilized in multiple CRISPR tasks, and second, the potential opportunities for leveraging existing AI methods to enhance predictive performance across different CRISPR tasks.

Out of 10 CRISPR tasks, 23 unique AI predictors have been utilized in on-target activity. Particularly, DL models such as CNN, RNNs, LSTMs, and ML models such as LR, RF, and XGBoost have been employed commonly as compared to other models. In spite of on-target activity prediction being a regression task, traditional models have not been explored properly such as ridge, lasso, and elastic regression. In terms of off-target activity prediction, RNN and LSTMs have been more commonly used. It is noteworthy to mention here that only for off-target activity prediction [117] the potential of language models is explored. Similarly, for CRISPR arrays, loci, editable gene target identification, cas proteins, and aca have witnessed development of multifarious predictive pipelines but only a few pipelines encompass DL models. In these tasks, CNNs, hybrid models, DL models with attention, and language models potential have not been explored yet.

### Experimental setting and evaluation strategies for CRISPR tasks

In the evaluation of AI predictors for CRISPR tasks, predictors are typically trained and tested in two different experimental settings: cross-validation [145] and independent testing [128]. In cross-validation, first the data is divided into *K* equal subsets. Then the predictor is trained on *K-1* subsets and tested on the remaining subset. This process is repeated k times to ensure each sample participates into model training and evaluation [145]. Independent testing, on the other hand, uses a separate dataset that was not involved in the training process which provides an unbiased evaluation of predictor performance. This approach helps to validate the predictor’s ability to generalize to new data and also ensures that the observed performance is not due to overfitting on the training data [128].

**Fig. 13:**
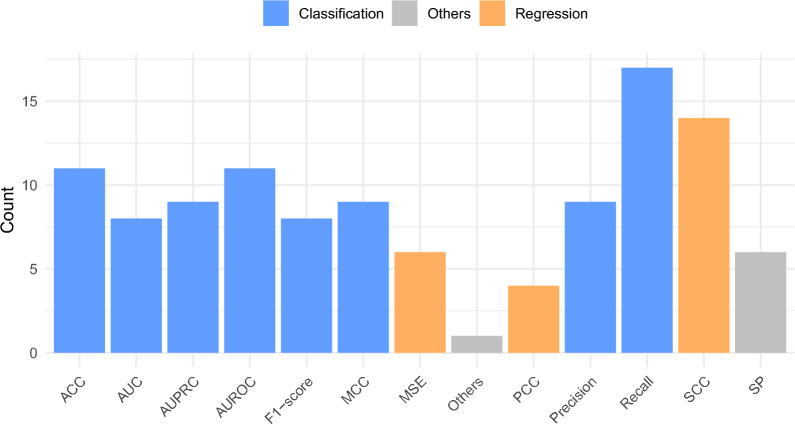
Count of evaluation measures used to assess the predictive performance of classifiers and regressors

In the realm of CRISPR tasks, along with these two experimental settings researchers have employed various evaluation metrics to measure predictor effectiveness. Table 16 and Fig. 13 show that 12 different evaluation measures have been utilized by existing studies. 8 out of 12 different evaluation measures have been used to evaluate classification predictors which include measures like accuracy (ACC) [138], precision [221], recall [187], specificity (SP) [35], F1-score [117], area under the ROC curve (AUROC) [78], area under the precision-recall curve (AUPRC) [139], and Matthews correlation coefficient (MCC) [109]. On the other hand, regression-based studies utilized 4 distinct evaluation measures namely, mean squared error (MSE) [172], Pearson correlation coefficient (PCC) [234], Spearman correlation coefficient (SCC) [234], and Kendall Tau [116].

For classification tasks, ACC measures the proportion of true results (both true positives and true negatives) among the total number of samples [138]. Precision is the ratio of correctly predicted positive samples to the total predicted positives [221]. Recall is the ratio of correctly predicted positive observations to all the observations in the actual class [187]. Specificity is the ratio of true negative predictions to the total number of actual negative instances [35]. F1 score is the weighted average of precision and recall and provides a balance between the two [117]. It is particularly useful when the class distribution is imbalanced. AUROC evaluates the ability of the predictor to distinguish between classes [78], and AUPRC focuses on the performance of the predictor for the positive class [139], especially important in datasets with class imbalance. MCC takes into account true and false positives and negatives, providing a balanced measure even if the classes are of different sizes [109].

For regression tasks, MSE measures the average of the squares of the errors [172]. The Pearson correlation coefficient assesses the linear correlation between predicted and actual values [234]. The Spearman coefficient measures the rank correlation between predicted and actual values [61, 234]. Kendall Tau is a statistic used to measure the ordinal association between two measured quantities which is useful to understand the strength and direction of association [116].

Although a plethora of evaluation measures exist for the performance evaluation of AI predictors, it is important to recognize that each evaluation measure has its pros and cons. For instance, metrics like ACC provide a straightforward measure of overall predictor correctness but may not account for class imbalances [80]. Meanwhile, regression metrics like MSE quantify prediction errors but may be sensitive to outliers, while correlation coefficients like Pearson and Spearman assess the strength and direction of relationships but may not capture all nuances of predictive accuracy [83].

It is essential to highlight that some studies did not use a sufficient number of evaluation metrics, which can result in potential issues in the evaluation of AI predictor. For instance, Rafid et al. [152] uses only AUROC, potentially overlooking precision and recall, while Yi et al. [223] focuses only on recall, which can lead to a high number of false positives if precision is not considered. Niu et al. [139] uses ROC, precision, recall, and AUPRC but omits metrics like the F1-score which provides a balanced view of performance.

Overall, it can be concluded that utilizing limited metrics can lead to an incomplete understanding of predictor performance, especially in imbalanced datasets. Therefore, employing a comprehensive evaluation approach is crucial. For classification predictors, combining metrics such as accuracy, precision, recall, F1-score, AUROC, and AUPRC captures various performance aspects, ensuring robustness across scenarios. For regression predictors, using MSE, Pearson, Spearman, and Kendall Tau evaluate both prediction error magnitude and the strength of relationships, offering a holistic view of predictive power and reliability. This comprehensive evaluation strategy is vital for advancing CRISPR research and developing effective gene-editing tools.

**Table 16:** Evaluation metrics used by the reviewed papers

	Study	Evaluation metrics
Targ.	[10]	average mutual score, hybrid score, microhomology score, vertical score
On-target Activity Pred.	[209]	SCC
	[152]	AUROC
	[61]	SCC
	[50]	PC, AUROC
	[103]	AUROC, SCC, PC
	[138]	AUROC, SN, SP, MCC, ACC
	[40]	SCC
	[61]	SCC
	[141]	PC
	[92]	SCC, nDCG, R-Precision, AUC
	[127]	ROC, AUC, Kolmogorov-Smirnov test
	[34]	SCC, MSE
	[234]	PCC, MSE, Steiger’s Test, SCC, ANOVA, Tukey’spost hoc test
	[219]	MSE, cosine similarity
	[231]	SCC, AUROC, Kolmogorov-Smirnov test
	[228]	SCC
	[232]	SCC, AUROC, PRAUC
Off-target Activity Pred.	[178]	ACC, Precision, Recall, F1-score, AUROC, AUPRC
	[78]	Validation Loss, ACC, AUPRC, AUROC
	[112]	Recall, Precision, ROC
	[139]	ROC, Precision, Recall, AUPRC
	[221]	AUPRC, AUROC, Recall, Precision
	[238]	PCC and AUPRC (for mutations)
	[117]	AUROC, AUPRC, F1 score, and MCC
	[232]	SCC, AUROC, PRAUC
	[187]	Recall, Precision, MCC, R-squared
	[172]	MSE, AUPRC
Acr/Activity Pred.	[109]	ACC, Precision, Recall, F1-score, MCC
	[63]	F1 score, ACC, AUC, Precision, Recall
	[35]	ACC, SN, SP, MCC, AUC
	[242]	PRE, SN, SP, F-score, ACC, MCC
	[57]	ROC, AUC, Precision, Recall, ACC
	[193]	SN, SP, ACC, F-Value, MCC, AUC
	[47]	-
	[132]	Precision, recall, ACC, F1-score, MCC
Mut.	[116]	MSE, AUC, PC, Kendall Tau
	[105]	AUC-ROC, Pearsons Coefficient
	[234]	
CR. Array	[37]	AUC, SN, SP
	[128]	SN, SP, learned evaluation function
	[157]	ACC
	[137]	AUC, Recall, Precision, F-1 score
Cas.	[145]	Adjusted balanced ACC, F-score with macro-averaging, MAE
	[235]	ACC, SP, SN, MCC
OP.	[217]	Recall
	[223]	Recall
ACA	[216]	Recall
C.-sys.	[145]	ACC, F1-score, SN
	[147]	ACC, F1-score

### Libraries and AI driven CRISPR applications source codes

This section compiles detailed information on open source predictors and the libraries they leverage in various CRISPR tasks. By providing this comprehensive overview, researchers can build upon existing tools, promote collaboration and advance the development of effective CRISPR prediction predictors.

Table 17 encompasses links to the 45 open source code repositories and respective libraries utilized by them. Among the 50 CRISPR-related studies, 40 have provided publicly accessible source code. Among these studies, 22 have utilized Python libraries such as TensorFlow and Keras [40, 61, 78, 92, 103, 105, 116, 117, 128, 138, 152, 187, 209, 219, 221, 228, 231, 232, 238]. Additionally, 15 studies have employed PyTorch [47, 63, 109, 112, 172, 178, 242]. Other ML libraries such as Scikit-learn, XGBoost, ViennaRNA, and BioPython have also been commonly integrated to design predictors [40, 61, 78, 92, 105, 112, 128, 138, 152, 187, 209, 219, 221, 232, 238].

A detailed analysis of these open source codes reveals that the majority of these tools have been developed using well-established libraries, promoting a standardized approach while also fostering innovation. This integration of well-established libraries contributes to the robustness and effectiveness of CRISPR prediction models within the research community.

The selection of a specific library for CRISPR tasks is inherently subjective and depends on factors such as the preferred development platform, the choice of prediction models, and the specific research questions at hand. Therefore, recommendations are made based on the variety of models and evaluation measures each library offers. For Python, TensorFlow and Keras are highly recommended due to their extensive support for DL models and user-friendly APIs [78, 221, 238]. PyTorch is also favored for its flexibility in model development and dynamic computational graphs [112, 172, 178]. Additionally, libraries such as Scikit-learn and XGBoost are valuable for more traditional ML approaches due to their comprehensive suite of algorithms and ease of integration into various workflows [47, 92]. Ultimately, selecting the right library aligned with individual research needs not only streamlines the development process but also enhances the overall reliability and effectiveness of CRISPR prediction models.

**Table 17:** Source links and libraries

Problem	Study	Source Code	Libraries
Editable target region ident.	[10]	Link	
Off-target activity prediction	[178]	Link	Pytorch
	[78]	Link	Tensorflow, keras, sklearn, gensim
	[112]	Link	Torch, sklearn
	[139]		
	[221]	Link	Tensorflow, keras, sklearn
	[238]	Link	Tensorflow, sklearn
	[117]	Link	Tensorflow, keras, sklearn
	[232]	Link	Tensorflow, keras
	[187]	Link	Tensorflow, keras
	[172]	Link	Sklearn, pytorch
On-target activity prediction	[209]	Link	Tensorflow, keras, sklearn, biopython, viennarna
	[50]	-	–
	[34]	-	–
	[234]	-	–
	[231]	Link	Sklearn, keras, tensorflow
	[127]	Link	Viennarna
	[234]	Link	
	[219]	Link	Tensorflow
	[92]	Link	Sklearn, xgboost
	[141]	Link1Link2	Sklearn
	[232]	Link	Keras, tensorflow
	[61]	Link	Keras, sklearn, tensorflow, biopython
	[40]	Link	Tensorflow, sklearn
	[152]	Link	Sklearn
	[103]	Link	Sklearn, keras, tensorflow
	[138]	Link	Sklearn, keras
	[228]	Link	Viennarna, biopython
Acr	[63]	Link	Bio, biolib, biopython, pytorch, sklearn, transformers
	[109]	Link	Torch, sklearn
	[242]	Link	Sklearn, lightgbm, xgboost, catboost
	[57]	Link	Sklearn
	[47]	Link	Sklearn, xgboost
	[35]	Link	–
CRIPSR Array	[128]	Link	Keras, sklearn, biopython, viennarna, hmmer, blast
	[157]	Link1Link2	Sklearn, biopython, xgboost
Mutations	[116]	Link	Mittens, keras, pytorch, tensorflow
	[105]	Link	Tensorflow, keras
Cas protein prediction	[187]	Link	Tensorflow, keras, sklearn
	[145]	Link	Sklearn, hmmer,
	[147]	Link	–
	[235]	Link	Keras, tensorflow, sklearn, biopython, prodigal, blast, hmmer, viennarna, xgboost
	[172]	Link	Torch, tensorflow, sklearn, xgboost
Operons	[217]	Link	Biopython
	[223]	Link	CRISPRCasFinder, psiblast+, blastn (NCIB), blastp (NCIB)
Aca	[216]	Link	VIBRANT, cctyper, diamond, hmmer, prodigal

### Performance values of AI-predictors in CRISPR

This section presents the predictive performance values of 37 AI predictors across 10 different CRISPR tasks, evaluated on 77 benchmark datasets. In-depth analyses of these predictors using evaluation measures such as precision, recall, F1-score, SCC, PCC, and AUC, offer insights into the strengths and weaknesses of various feature extraction and classification methods specific to different CRISPR tasks. This comprehensive analysis aids in selecting the most suitable classifiers and feature extraction methods, optimizing experimental design. Additionally, it identifies tasks for improvement, promoting innovation in AI predictor development and facilitating cross-disciplinary research.

Table 18 presents performance values of 37 predictors across 8 different CRISPR tasks namely, i.e., on/off-target activity prediction, CRISPR array identification, Cas proteins prediction, acr and aca proteins identification, acr proteins activity prediction, and gene editing outcome prediction. It encompasses predictive performance values in terms of 13 distinct evaluation measures ACC, SN, SP, AUCROC, FP, F1, MCC, AUPRC, Cohen’s Kappa, R$^{2}$, MSE, SCC, and PCC. Two different types of trends can be observed here i.e., which feature extraction and AI method performs well in a single task and secondly which specific set of feature extraction and AI method performs well across multiple CRISPR tasks.

For CRISPR array identification, the predictor by [37] demonstrates superior performance. This approach uses randomly initialized embeddings for representation learning and an LSTM classifier, achieving high predictive performance: ACC: 94.58, SN: 91.99, SP: 97.17, and AUCROC: 98.72 in a 5-fold cross-validation setting. The use of LSTM classifiers is particularly effective here due to their ability to capture long-range dependencies in sequence data, which is crucial for identifying complex patterns in CRISPR arrays.

In the domain of CRISPR loci classification, the predictor by [157] performs exceptionally well with a k-mer counts (4-mer) feature extraction method and a gradient boosting decision trees classifier, yielding a median accuracy (ACC) of 98.6 and a false positive count (FP) of 28 (0.4). K-mer based features effectively capture sequence composition, while gradient boosting classifiers leverage these features to distinguish between different loci types. Additionally, the predictor by [137], which utilizes features such as length, GC content, palindromic index, and k-mers, combined with multivariate logistic regression, XGBoost, and OVA XGBoost classifiers, achieves an F1-score of 0.97. This combination is effective because it integrates both sequence and structural information, enhancing predictive accuracy.

For Cas protein prediction, two approaches stand out. The predictor developed by [220] uses di-peptide composition for feature extraction and an SVM classifier, achieving metrics of SN: 83.71, SP: 86.77, ACC: 84.84, MCC: 0.70, and AUCROC: 0.8945. Di-peptide composition captures essential biochemical properties of proteins, while SVM classifiers effectively separate classes in high-dimensional spaces. Another significant predictor is by [235], which employs a wide range of features including AAC, ASDC, CKSAAP, DDE, QSO, DPC, PSSM, AATP, Pse-PSSM, TTri-gram-PSSM, CTD, CTDC, CTDT Transition, and UniRep. This method uses a stacked ML approach with baseline classifiers such as LGBM, RF, ERT, GBDT, and XGBoost, and a meta classifier SVM. The performance on the Cas300 dataset is outstanding with ACC: 97.28, MCC: 0.944, SN: 97.71, and SP: 96.31. The combination of diverse peptide features and stacked ML models is effective because it captures various aspects of protein sequences, enhancing prediction robustness.

In the area of acr protein prediction, the work by [109] shows that using RaptorX-based structure and solvent accessibility features, transformer embeddings from ESM-1b, POSSUM, and one-hot encoding, processed through CNNs and FCNs, achieves strong results. For instance, AcrNet-1 achieves ACC: 0.7979, P: 0.8363, SN: 0.6810, F1: 0.7505, and MCC: 0.5924, while the combined dataset achieves ACC: 0.9442, P: 0.9471, SN: 0.9409, F1: 0.9418, and MCC: 0.8883. These methods work well because they combine structural, sequence, and embedding features, providing a comprehensive representation of proteins. Similarly, the predictor by [35], which uses PSSM-based features and an ML ensemble classifier, achieves high metrics with 5-fold cross-validation, including SN: 0.923, SP: 0.877, ACC: 0.881, and AUC: 0.952. The success of PSSM-based features and ensemble classifiers lies in their ability to capture evolutionary information and aggregate multiple models’ strengths.

In the domain of gene editing outcome prediction, the study by [116] uses GloVe and positional encoding with BiLSTM and attention mechanisms, achieving an MSE of 0.000164 and high AUC and PCC values for various editing outcomes. Word embeddings like GloVe capture contextual meaning, while attention-based BiLSTM models are adept at handling dependencies and variations in gene editing outcomes.

The prediction of on/off-target activity presents unique challenges due to significant variability in datasets and predictive models. Each study often uses different datasets, making direct comparisons difficult and limiting the generalizability of findings. For instance, [178] utilizes the [29] dataset OHE and LSTM classifiers, achieving high performance metrics such as ACC: 0.997 and AUCROC: 0.990. While the use of publicly available datasets like [29] enhances reproducibility, differences in cell types and experimental conditions between studies still pose challenges for direct comparison. In another example, [78] utilizes word2vec embeddings and BiLSTM on the K562 cell line dataset, achieving ACC: 99.40 and AUPRC: 86.67. While embeddings capture semantic information effectively, the results are highly specific to the K562 dataset, complicating generalizability. The study by [139] uses multiple datasets, including CIRCLE-seq [182], PKD (II/1) [43], and others, with LSTM and OHE, yielding an AUROC of 0.976. The use of diverse datasets aims to improve generalizability but introduces variability in experimental conditions, making uniform assessment challenging. Similarly, [221] employs several datasets, such as I/1 [182] and II/2 [60], with BiLSTM and embedding, achieving an AUPRC of 58.58 and AUCROC of 98.74. The inclusion of diverse datasets enhances robustness but complicates performance evaluation due to varying dataset characteristics.

**Table 18:** Performance values of 39 different predictors across 76 different benchmark datasets related to 10 different CRISPR tasks

	Name	Author, year	Dataset	Rep.Learning	Classifier	Performance
B. Cls.	CRISPR Array	[37]	[37]	Randomly initialized Embeddings	LSTM	5-fold: ACC: 94.58, SN:91.99, SP:97.17, AUCROC:98.72
		[128]				
MC Cls.	CRISPR loci	[157]	[157]	k-mer counts (4-mer)	gradient boosting decision trees	IND: med ACC: 98.6, FP: 28 (0.4)
		[137]	[137]	Length, GC content, palindromic index, k-mers	multivariate logistic regression, XGBoost, OVA XGBoost	OVA (one-vs-all) XGBoost: F1: 0.97
B.Cls	Cas Protein	[220]	[220]	Di-peptide composition	SVM	ind: SN: 83.71, SP: 86.77, ACC: 84.84, MCC: 70.0, AUCROC: 89.45
		[235]	[235]	AAC, ASDC, CKSAAP, DDE, QSO, DPC, PSSM, AATP, Pse-PSSM, TTri-gram-PSSM, CTD, CTDC, CTDT Transition, UniRep	Stacked ML: Baseline Classifiers (LGBM, RF, ERT, GBDT, XGBoost), Meta Classifier: SVM	Cas300: ind: ACC: 97.28, MCC: 0.944, SN: 97.71, SP: 96.31
						Cas300: ind: ACC: 94.07, MCC: 0.866, SN: 96.61, SP: 91.52
	Anti-CRISPR proteins	[109]	AcrNet-1	RaptorX based structure and solvent accessibility features , Transformer embeddings from ESM-1b, POSSUM, and one hot encoding	CNNs and FCNs	5-fold: ACC: 79.79, P: 83.63, SN: 68.10, F1: 75.05, MCC: 59.24
			Gussow			5-fold: ACC: 95.43, P: 97.62, SN: 93.60, F1: 95.53, MCC: 91.01
			AcrNet-2			5-fold: SP: 95.77, ACC: 89.42, P: 48.95, SN: 76.00, F1: 63.27, MCC: 52.39
			AcrNet-3			5-fold: SP: 95.38, ACC: 77.36, P: 31.06, SN: 73.72, F1: 44.32, MCC: 36.55
			Combined (1,2,3)			5-fold: ACC: 94.42, P: 94.71, SN: 94.09, F1: 94.18, MCC: 88.83
		[35]	AcrPred	DPC, CTD, PSSM, PSSM-composition, DPC-PSSM, PSSM-AC, RPSSM	ML ensemble	5-fold: SN: 92.3, SP: 87.7, ACC: 88.1, AUC: 95.2
		[242]	PreAcrs	PSSM-AC, RPSSM and SSA	LR ensemble of SVM, KNN, MLP, LR, RF, XGBoost, LightGBM, CatBoost	IND: P: 98.6, SN: 79.5, SP: 98.9, F1: 88.1, ACC: 89.2, MCC: 79.9, AUC: 97.2, AUPRC: 97.6
		[57]	[57]	self-targeting genomes, annotated protein fractions in directions, protein lengths, presence of HTH domains downstream, directon size, and protein hydrophobicity	Extra Trees	IND: AUC-ROC: 83.0, 15-fold: 93.0
		[193]	[193]	PSSM-Composition, DPC-PSSM, PSSM-AC, and RPSSM	SVM	IND: SN: 90.9, SP: 85.6, ACC: 88.2, F-value: 88.3, MCC: 76.5
		[47]	[47]	AAC, Grouped Dimer and Trimer Frequency Counts	XGBoost	-
				QSO, DPC.		
B.Cls.	Off-target Activity Prediction	[178]	[29]	OHE	LSTM (Best)	IND: ACC:99.7, P: 73.4, SN: 61.1, F1: 66.7, AUCROC; 99.0, AUPRC; 72.11
		[78]	[29]	W2vec embedding	BiLSTM	K562: IND: ACC: 99.40, AUPRC:86.67, AUROC:99.61
						HEK293T: IND: ACC: 99.40, AUPRC:66.20 AUROC:99.21
		[139]	CIRCLE-seq [182]	OHE	LSTM	5-fold: AUCROC: 97.6, AUORC: 48.0
			PKD (II/1) [43]			-
			Digenome PDH (II/2) [60]			-
			II/3 SITE			-
			II/4 [181]			Train (II-4), Test (II-5): IND: AUROC: 99.1, AUPRC: 31.9,
			II/5 [90]			Train ( CIRCLE, PCR, Digenome, SITE, and II-4), Test (II-5): (AUROC = 99.3, AUPRC = 29.7), “Train (CIRCLE, PKD, PDH, GUIDE-I), Test: (AUROC = 98.9, AUPRC = 25.4)”
						Train (PKD, PDH, SITE, GUIDE-I), Test: (AUROC = 99.1, AUPRC = 31.9)
						Train (CIRCLE), Test: (AUROC = 99.3, AUPRC = 17.3)
						Train (CIRCLE, PKD, PDH, SITE, GUIDE-I), Test (AUROC = 99.1, AUPRC = 31.2)
						Train (SITE), Test: (AUROC = 99.1, AUPRC = 25.01)
						Train (PKD, PDH, GUIDE-I), Test: (AUROC = 99.2, AUPRC = 26.5)
						Train (CIRCLE, SITE), Test: (AUROC = 99.4, AUPRC = 22.0),“Train (CIRCLE, PKD, PDH, SITE, GUIDE-I), Test: (AUROC = 99.3, AUPRC = 13.1)”
			II/6 [115]			Train (PKD, PDH, SITE, GUIDE-I), Test: (AUROC = 99.8, AUPRC = 18.4)
						Train (CIRCLE, PKD, PDH, SITE, GUIDE-I), Test: (AUROC = 99.92, AUPRC = 14.3)
						Train (PKD, PDH, GUIDE-I), Test: (AUROC = 99.94, AUPRC = 15.0)
						Train (CIRCLE, PKD, PDH, SITE, GUIDE-I), Test: (AUROC = 99.6, AUPRC = 11.9)
		[221]	I/1 [182]	Embedding	BiLSTM	5-fold: AUPRC: 58.58, AUCROC: 98.74
			I/2 [115]			-
			II/1 [43]			Train (I/1,II/5, I/2) AUCROC:87.31; AUPRC: 53.21
			II/2 [60]			Train (I/1,II/5, I/2) AUCROC:87.31; AUPRC: 53.21
			II/3 [19]			AUPRC:79.6
			II/4 [181]			-
			II/5 [90]			-
			II/6 [115]			-
			K562 [29]			AUCROC: 99.79 AUPRC: 80.49
			HEK293T [29]			AUCROC:98.79, AUPRC:78.39
		[117]	K562[29]	Positional encoding and OHE	BERT	AUROC: 99.9, PRAUC: 97.6, F1: 88.9, MCC: 88.6
			HEK293T [29]			AUROC: 97.0, PRAUC: 52.2, F1: 33.9, MCC: 40.4
			II/4 [181]			IND: Train (HEK293t, K562, II5 (combined)), AUROC: 99.8, PRAUC: 63.0, F1: 48.0, MCC: 53.2
			II/5 [90]			AUROC:0.998, PRAUC: 0.444, F1 score: 0.333, MCC: 0.344
			II/6 [115]			AUCROC: 99.7, AUPRC: 44.4
			I/1 [182]			AUROC: 98.7, PRAUC: 76.4, F1: 64.6, MCC: 65.5
			I/2 [115]			AUROC: 99.8, PRAUC: 64.1, F1: 56.4, MCC: 59.6
		[232]	K562 [29]	Embedding	CNN + attention	AUROC: 99.4, PRAUC: 81.6
			HEK293T [29]			IND: AUROC: 97.3, PRAUC: 79.0
		[187]	I/1[182] (negative data doesnot have Cas-cleavage)	OHE	CNN+BiLSTM	IND: P: 91.0 SN: 87.0 F1: 89.0, MCC: 80.0, Cohen’s Kappa: 0.77, R: 0.71
		[172]	CrisprSQL [171]	OHE, and nucleosome+epigenetic features	CNN+BiGRU	AUCROC: 99.5, AUPRC: 78.2
		[238]	II [115]	OHE	CNN	AUPRC: 26.2
			II/5 [90]			AUPRC: 36.4
			II [115]			AUPRC: 32.4
		[112]	II/4 [181]	OHE	CNN	5-fold: AUCROC:98.0, AUPRC:32.0
			II/2 [60]			5-fold: AUC-ROC: 98.0, AUPRC:42.0
	Gene editing outcome	[116]	Lindel	Glove and Positional encoding	BiLSTM + Attention	IND: MSE: 0.000164
			Forecast			Deletion frequency (AUC: 0.91, PCC: 0.53) 1 bp Insertion frequency (AUC: 0.94, PCC: 0.86) 1 bp Deletion frequency (AUC: 0.83, PCC: 0.70) 1 bp Frameshift frequency (AUC: 0.77, PCC: 0.43) 2 bp Frameshift frequency (AUC: 0.73, PCC: 0.46) Frameshift frequency (AUC: 0.69, PCC: 0.26)
			CROTON			Deletion frequency (AUC: 84.6, PCC: 0.7), 1 bp Insertion frequency (AUC: 88.1, PCC: 76.9)
Regression	On-target Activity	[209]	SpCas9-HF (ESP) [190]	OHE + Embedding	CNN, RNN+Attention	IND: (SCC: 0.867; Mean: $3.71\times 10^{-3}$)
			SpCas9-HF1 [190]			IND: (SCC: 0.867; Mean: $2.65\times 10^{-3)}$
			WT-SpCas9 [190]		IND: (SCC: 0.872; Mean: $2.55\times 10^{-3)}$	
		[40]	Chari	OHE	CNN	SCC: 0.49
			Wang/Xu			SCC: 0.69
			Doench Mouse			SCC: 0.51
			Doench Human			SCC: 0.23
			Hart HCT116			SCC: 0.55
			Moreno-Mateos			SCC: 0.19
			Gandhi			SCC: 0.36
			Farboud			SCC: 0.60
			Varshney			SCC: 0.35
			Gagnon			SCC: 0.35
Classification		[152]	Hart HCT116	Position Independent Features (PIF), Position Specific Features (PSF), and n-Gapped Di-nucleotides (nGD).	SVM	AUCROC: 87.9
			Chari 293T			AUCROC: 44.4
			Hart HeLA			AUCROC: 79.7
			Xu HL60			AUCROC: 75.9
Classification		[50]	Hart HCT116	Sequence and epigenetic features based OHE	GAN + CNN + DNN	AUC-ROC: 0.9817
			Chari 293T			AUC-ROC: 99.62
			Hart HeLA			AUC-ROC: 97.23
			Xu HL60			AUC-ROC: 98.42
Regression		[50]	Hart HCT116			PCC: 0.6696
			Chari 293T			PCC: 0.7417
			Hart HeLA			PCC: 0.6247
			Xu HL60			AUCROC: 75.9 PCC: 0.5913
Regression		[103]	Hart HCT116	Sequence and epigenetic features based OHE	CNN	5-fold: SCC: 0.6548
			Chari 293T			5-fold: SCC: 0.7352
			Hart HeLA			5-fold: SCC: 0.6397
			Xu HL60			5-fold: SCC:0.5473
Classification		[103]	Hart HCT116			5-fold: AUCROC: 97.32
			Chari 293T			5-fold: AUCROC: 99.65
			Hart HeLA			5-fold: AUCROC: 97.14
			Xu HL60			5-fold: AUCROC: 97.06
		[138]	Glycine	OHE	CNN	ACC: 82.43, AUCROC: 85.29, SE: 99.83, SP: 64.25, MCC: 67.91
			Zea			ACC: 81.26, AUCROC: 80.94, SE: 81.83, SP: 81.66, MCC:71.56
			Sorghum			ACC: 78.25, AUCROC: 83.06, SE: 79.00, SP: 77.50, MCC: 55.27
			Triticum			ACC: 87.49, AUCROC: 78.95, SE: 96.47, SP: 77.52, MCC: 68.26
		[232]	SpCas9-HF (ESP) [190]	Embedding	CNN+Attention	10-fold: SCC: 0.850
			SpCas9-HF1 [190]			10-fold: SCC: 0.853
			WT-SpCas9 [190]			10-fold: SCC: 0.848
			Sniper-Cas9 [88]			10-fold: SCC: 0.931
			xCas9 [88]			10-fold: SCC: 0.864
Regression		[141]	Hart HCT116, Chari 293T, Hart HeLA, Xu HL60,	Raw values, one-hot encoding, quantum chemical properties (QCT), and k-mers	iRF	R2: 0.229489979, PCC: 0.486193
			Combined: Hart HCT116, Chari 293T, Hart HeLA, Xu HL60, and Donech HM			R2: 0.211671332, PCC: 0.4964907
			Donech HM			R2: 0.389120714, PCC: 0.6525512
			Combined: Guo, Hart HCT116, Chari 293T, Hart HeLA, Xu HL60, and Donech HM			R2: 0.486194, PCC: 0.6972761, [E.coli 0.504], [H.sapien 0.491]
			Guo E.coli			R2: 0.249, PCC: 0.5019173
Regression		[234]	Hart HeLa-Lib1	OHE	BiLSTM	10-Fold: SCC: 0.438
			Hart HCT116-Lib1			10-Fold: SCC: 0.479
			Hart RPE			10-Fold: SCC: 0.375
			Hart HeLa-Lib2			10-Fold: SCC: 0.493
			Doench A375			10-Fold: SCC: 0.471
			Xu HL60			10-Fold: SCC: 0.622
			Xu KMB7			10-Fold: SCC: 0.644
			Chari 293T			10-Fold: SCC: 0.52
			Doench MM			10-Fold: SCC: 0.645
			Doench MOLM13			10-Fold: SCC: 0.705
			Endo-293T			10-Fold: SCC: 0.652
			Endo-H1			10-Fold: SCC: 0.468
			Endo-K562			10-Fold: SCC: 0.503
		[219]	CRISPRoff-tiling	OHE	CNN	AUC: 87.9, SCC:0.58-0-60
			CRISPRoff-genome			AUC: 68.7
			CRISPRi-activityscore			AUC: 71.6
			CRISPRi-K562			AUC. 83.3
			hCRISPRa-V2			AUC: 71.6
			CRISPRi-genome			AUC: 61.9
			hCRISPRi-V2			AUC: 60.9
Regression		[231]	Combined: Hart HCT116, Chari 293T, Hart HeLA, Xu HL60	OHE of sequence and epigenetic features	CNN-SVR	10-Fold: HCT116 (SCC: 0.719, AUCROC: 0.933) HEK293T (SCC: 0.807, AUCROC: 0.983) HELA (SCC: 0.699, AUCROC: 0.933) HL60 (SCC: 0.589, AUCROC: 0.934)
			Hart HCT116			leave one cell out: SCC: 0.719, AUCROC: 93.3
			Chari 293T			leave one cell out: SCC: 0.807, AUCROC: 98.3
			Hart HeLA			leave one cell out: SCC: 0.699, AUCROC: 93.3
			Xu HL60			leave one cell out: SCC: 0.589, AUCROC: 93.4
		[228]	Train: Combined (HTCas9 Kim, Xiang-gRNA)	GC, k-mer frequencies (k=1, 2, 3), poly-T segments features, melting temperatures , and minimum free energy	LightGBM regression	-
			Chari 293T			SCC: 0.466
			Doench Hs			SCC: 0.704
			Doench Mm			SCC: 0.603
			Doench azd-hg19			SCC: 0.413
			Hart HCT1162Lib1			SCC: 0.479
			Hart HeLaLib1			SCC: 0.45
			Hart HeLaLib2			SCC: 0.503
			Hart RPE1			SCC: 0.355
			Xu HL60			SCC: 0.604
			Xu KBM7			SCC: 0.622
			Gagnon			SCC: 0.29
			Moreno-Mateos			SCC: 0.181
			Varshney			SCC: 0.327
	Cas. Sys.	[145]	-	HMM profiles of cassettes	SVM, HMM, CART, ERT	IND: ACC: 98.56, 50-fold: CART Mean F1: 0.97, SVM Mean F1: 0.98, ERT Mean F1: 0.99
	CrRNA	[147]	[147]	-	KNN	F1: 89.0, and ACC: 92.3

Finally, here we make some recommendations related to CRISPR tasks and the use of feature extraction methods and AI predictors. Feature extraction methods such as k-mer counts, PSSM, structural features from RaptorX, OHE, and random and transformer embeddings consistently show high performance across various CRISPR tasks. These methods should be prioritized because they capture essential biological and sequence-specific information. Ensemble methods (e.g., gradient boosting, XGBoost), LSTM/BiLSTM, and attention-based neural networks prove effective due to their ability to handle complex patterns and integrate diverse features.

### Discussion

The integration of AI has markedly enhanced the efficiency and accuracy of CRISPR systems, particularly in the identification of target sites, prediction of off-target effects, and optimization of gene editing outcomes. Our analysis reveals that while AI predictors have been developed for 10 different CRISPR tasks, there is a notable emphasis on the prediction of on/off-target activities and acr proteins. These tasks are critical due to their direct impact on the specificity and safety of CRISPR-based genome editing. The ability to predict and minimize off-target effects is paramount in ensuring the precision and efficacy of CRISPR interventions [29, 182]. Despite these advancements, there remains a substantial need for further innovation to address the complexities inherent in genetic diseases and the variability in individual genetic makeup.

In terms of 10 different CRISPR tasks, 80 distinct benchmark datasets have been developed i.e., CRISRP arrays: 2, CRISPR loci:2, Cas-proteins: 2, acr proteins: 9, acr proteins activity: 2, off-target activity: 15, on-target activity: 39, gene editing outcomes: 4 and others: 5. In the current research landscape of AI in CRISPR tasks, the majority of studies rely on public datasets rather than proprietary in-house data. This trend ensures fair performance comparisons between new predictors and existing models. Despite the vast array of available datasets, only a few are commonly utilized. This heterogeneity among different studies for a single application can result in models that perform well on certain datasets but poorly on others, limiting their applicability in real-world scenarios. For instance, off-target activity prediction models like DeepCRISPR has been evaluated on 2 cell lines datasets, such as K562 and HEK293T [29]. Other off-target activity prediction models, such as RCrispr, have been evaluated on datasets like CIRCLE-seq [182] and PKD [43]. MisIndel used datasets like I/1 [182], I/2 [115], and II/3 [19]. Models like piCRISPR [171] and hybrid multitask [182] have also limited cohort of datasets such as CrisprSQL and I/1. On-target activity prediction models have been evaluated similarly on limited number of datasets. For example, AttCrispr, was evaluated on datasets like SpCas9-HF1 and WT-SpCas9 [190], and CRISPRPred was evaluated on Hart HCT116 and Chari 293T [152]. GanOnTarget used datasets such as Hart HCT116, Chari 293T, Hart HeLA, and Xu HL60 [50]. Models like CnnXg [103] and quantum [141] have utilized a few datasets including Hart HCT116, Chari 293T, Hart HeLA, and Xu HL60. Similar trends exist for other CRISPR tasks where benchmarking is not conducted properly across a broad cohort of datasets, leading to inconsistent performance comparisons and the development of less powerful predictors.

To address these challenges, it is recommended to develop and use standardized, publicly available benchmark datasets and establish consistent evaluation protocols. This would enable more reliable comparisons and enhance reproducibility. By addressing this issue, the field can move towards more robust and generalizable predictive models for CRISPR tasks, advancing both research and clinical applications.

In developing AI predictors for CRISPR tasks, the selection of feature extraction methods and classifiers or regressors should be done carefully, as these choices can significantly impact the model’s performance and interpretability. Inappropriate feature extraction methods may fail to capture crucial genomic information, while suboptimal classifiers or regressors can lead to poor prediction accuracy and generalization issues. In terms of CRISPR tasks, methods such as k-mer counts, OHE, and advanced embedding techniques have proven effective in capturing the complex details of DNA, RNA, and protein sequences. However, the potential of 29 different types of embedding methods has yet not been explored in CRISPR tasks, such as DANE [106], DeepWalk [126, 136, 198, 241], ELMo [81, 215, 237], FastText [97, 180], GATNE [224], GEMSEC [18], GraRep [8, 26, 202], MetaGraph2Vec [46], HAKE [191], HIN2Vec [205], HOPE [204, 239], Laplacian eigen maps [3], LINE [173, 192, 198], Locally linear embedding [3], Mashup [188, 206], Node2Vec [3, 104, 188], OPA2Vec [142], Random Watcher-Walker (RW2) [120], RotatE [20, 150, 191], RWR [176], SDNE [146, 198], SocDim [202, 212], Struc2Vec [28, 201], SVD [104, 240], Topo2Vec [121], TransE [185], and Graph2vec [135]. Moreover, the effectiveness of 19 distinct language models in CRISPR tasks remains untested. i.e., ALBERT [158, 218, 229], AlphaFold [52, 71, 73, 160, 196], AlphaFold2 [119, 156], BERT [102, 110, 129, 158, 160, 229], BigBird [33], ELECTRA [7, 229], ESM-1 [71, 124, 211], ESM-2 [118, 156, 211, 215], GPT [74, 82, 168, 179], Graph Transformer Network [107], Heterogeneous Graph Transformer [243], IgFold [125], LongFormer [33], RoBERTa [102, 158], T5 [49, 65, 211, 227], Transformer [33, 154, 169, 203], Transformer-XL [30], ULMFiT [123], Vision Transformer [81], and XLNet [229].

The evaluation of AI-driven CRISPR models has predominantly relied on a range of evaluation measures, including accuracy, precision, recall, and the area under the ROC curve. These metrics provide a comprehensive assessment of model performance, but the variability in evaluation strategies across studies underscores the necessity for standardized usage of evaluation measures. Such standardization would facilitate the comparison of different models and accelerate the development of accurate and better CRISPR applications [139, 209].

In summary, the integration of AI with CRISPR technology holds immense promise for advancing genetic research and therapy. To fully harness this potential, future research must focus on developing more interpretable AI predictors, standardizing evaluation metrics, and creating comprehensive benchmarking datasets. By addressing these challenges, researchers can enhance the precision, safety, and effectiveness of CRISPR-based interventions, paving the way for groundbreaking advancements in genetic medicine.

This review paper has several limitations, particularly in its coverage of technical aspects essential for understanding core AI concepts. Notably, it lacks an in-depth discussion on representation learning methods, which are crucial for converting CRISPR-related biological data into formats compatible with AI models, as highlighted in some existing studies [1, 133]. These gaps reduce the paper’s utility for researchers seeking to develop concepts about AI models and representation learning methods for CRISPR-related applications.

## Data Availability

The datasets during and/or analyzed during the current study are available from the corresponding author upon reasonable request.
